# Repulsive guidance molecules lock growth differentiation factor 5 in an inhibitory complex

**DOI:** 10.1073/pnas.2000561117

**Published:** 2020-06-23

**Authors:** Tomas Malinauskas, Tina V. Peer, Benjamin Bishop, Thomas D. Mueller, Christian Siebold

**Affiliations:** ^a^Division of Structural Biology, Wellcome Centre for Human Genetics, University of Oxford, OX3 7BN Oxford, United Kingdom;; ^b^Department of Molecular Plant Physiology and Biophysics, Julius-von-Sachs Institute, University of Würzburg, 97082 Würzburg, Germany

**Keywords:** TGFβ/BMP signaling, Repulsive guidance molecule, cell surface receptor, structural biology, Neogenin

## Abstract

Repulsive guidance molecules (RGMs) are cell surface proteins that regulate the development and homeostasis of many tissues and organs, including the nervous, skeletal, and immune systems. They control fundamental biological processes, such as migration and differentiation by direct interaction with the Neogenin (NEO1) receptor and function as coreceptors for the bone morphogenetic protein (BMP)/growth differentiation factor (GDF) family. We determined crystal structures of all three human RGM family members in complex with GDF5, as well as the ternary NEO1–RGMB–GDF5 assembly. Surprisingly, we show that all three RGMs inhibit GDF5 signaling, which is in stark contrast to RGM-mediated enhancement of signaling observed for other BMPs, like BMP2. Despite their opposite effect on GDF5 signaling, RGMs occupy the BMP type 1 receptor binding site similar to the observed interactions in RGM–BMP2 complexes. In the NEO1–RGMB–GDF5 complex, RGMB physically bridges NEO1 and GDF5, suggesting cross-talk between the GDF5 and NEO1 signaling pathways. Our crystal structures, combined with structure-guided mutagenesis of RGMs and BMP ligands, binding studies, and cellular assays suggest that RGMs inhibit GDF5 signaling by competing with GDF5 type 1 receptors. While our crystal structure analysis and in vitro binding data initially pointed towards a simple competition mechanism between RGMs and type 1 receptors as a possible basis for RGM-mediated GDF5 inhibition, further experiments utilizing BMP2-mimicking GDF5 variants clearly indicate a more complex mechanism that explains how RGMs can act as a functionality-changing switch for two structurally and biochemically similar signaling molecules.

## Significance

Repulsive guidance molecules (RGMs) are cell surface proteins that control processes ranging from brain development to iron metabolism. Dysregulation of RGM function leads to severe diseases. RGMs can bind to the cell guidance receptor Neogenin (NEO1) and act as coreceptors for the bone morphogenetic proteins (BMPs). Here, we determined high-resolution structures of all three human RGMs in complex with the BMP ligand growth differentiation factor 5 (GDF5) and the ternary complex of GDF5 with RGM and NEO1. Our structural and functional analyses reveal that RGM competes with the GDF5 receptor. This study shows that RGMs can act as inhibitors of a BMP ligand (GDF5), and not always as activators of BMP signaling, as previously reported.

The 20 members of the bone morphogenetic protein (BMP)/growth differentiation factor (GDF) morphogens comprise the largest group of the transforming growth factor-β (TGF-β) superfamily (1). They play essential roles in development and homeostasis of organisms (1). Secreted BMP ligands initiate signaling by binding to two types of receptor serine/threonine kinases: the BMP type 1 (BMPR1) and type 2 (BMPR2) receptors. Binding of both receptor types triggers phosphorylation of BMPR1 by constitutively active BMPR2 (2), which in turn triggers signaling via the SMAD1/5/8 transcription factors. GDF5 exhibits a defined BMPR1 specificity. Missense mutations in GDF5 or its BMPR1 receptor BMPR1B (ALK6) cause similar skeletal malformations (e.g., chondrodysplasia and brachydactyly), suggesting that GDF5 signaling is coupled to BMPR1B (3–6). Biochemical analyses revealed high-affinity binding between GDF5 and BMPR1B and a 10-fold lower affinity for BMPR1 receptor BMPR1A (ALK3) (7). GDF5 signaling via BMPR1A seems highly cell-dependent, in which GDF5 can either act as agonist (i.e., BMP2-like) or as antagonist (suppressing signaling by other BMPs) (8).

Repulsive guidance molecules (RGMs) are glycosylphosphatidylinositol (GPI)-anchored cell surface glycoprotein coreceptors for BMP/GDF morphogens and were shown to potentiate signaling of at least BMP2 and BMP6 (9–11). There are three members in humans: RGMA, RGMB (DRAGON), and RGMC (Hemojuvelin or HFE2). They play roles in cell migration, differentiation, and systemic iron metabolism (12, 13), while dysfunction is linked to severe diseases, such as multiple sclerosis and blood disorders (14, 15). RGMs can bind with high affinity to a subset of BMP ligands, as well as to the Neogenin receptor (NEO1). Structural analysis of RGMs and their complexes with NEO1 and BMP2 revealed a common RGM architecture comprising an N-terminal domain (RGM_ND_) essential for BMP binding and a C-terminal domain (RGM_CD_) sufficient for NEO1 binding (13, 16, 17). Importantly, RGMB can physically bridge NEO1 and BMP2, suggesting a functional link between these two pathways (17). Such a link has been suggested in hepatocytes, where NEO1-deficient mice exhibit iron overload, low levels of Hepcidin (a regulator of iron homeostasis), and reduced BMP6 signaling in liver (18). Similarly, RGMC missense mutations or RGMC-deficient mice exhibit reduced Hepcidin expression and iron overload (19–21). Recently, it has been shown that BMP2 controls iron homeostasis independent of BMP6 (22), suggesting functional ties between BMP2, BMP6, and NEO1. Moreover, NEO1-deficient mice showed defective bone formation and impaired differentiation of chondrocytes (23). Similarly, mutations in GDF5 alter the length and number of bones in the limbs of mice (24) and cause limb shortening in humans (4), suggesting that NEO1 and GDF5 might signal via the same pathway in chondrocytes. These functional ties between NEO1 and BMP/GDF signaling pathways are evolutionarily conserved, since UNC-40, a single NEO1 homolog in *Caenorhabditis elegans*, promotes BMP signaling via the RGM ortholog DRAG-1 (25).

We previously showed that RGM occupies the type 1 receptor binding site on BMP2 (17), raising two questions. First, how can RGMs potentiate BMP2/BMP6 signaling although they could compete with BMPR1 binding? Second, can RGMs inhibit instead of potentiate signaling induced by other BMPs/GDFs? Here, we show that GDF5 can bind to all RGMs with high affinity. However, in contrast to the closely related BMP2, all RGMs inhibit GDF5 signaling in cellular assays. The presence of GDF5 and BMP2 at the same time leads to neutralization of both RGM-mediated activation of BMP2 and inhibition of GDF5 signaling. To unravel the structural basis of how GDF5 signaling is controlled by RGMs and NEO1, we determined crystal structures of binary RGM–GDF5 complexes and the ternary NEO1–RGMB–GDF5 complex. A structural comparison to previously determined BMP2 complexes, combined with affinity measurements, suggests that RGMs can either activate or inhibit the BMP/GDF ligands depending on their interaction determinants.

## Results

### Membrane-Bound RGMs Inhibit GDF5 Signaling in Cellular Assays.

**Fig. 1.**
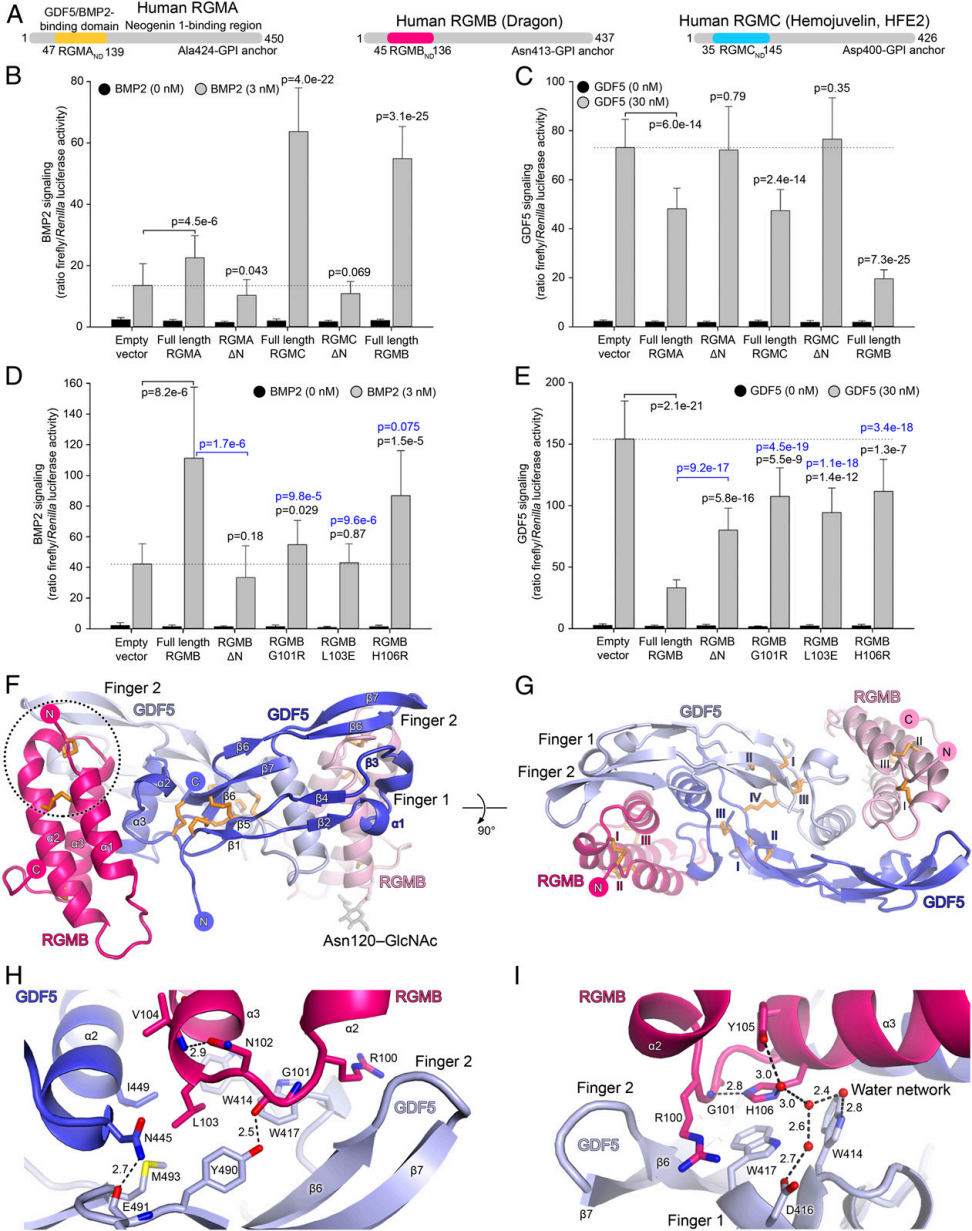
Cellular assay for RGM–BMP2/GDF5 signaling and the crystal structure of the RGMB–GDF5 complex at 1.7 Å resolution. (*A*) Domain organization of human RGMs with the N-terminal domains (RGM_ND_s) indicated. (*B* and *C*) BMP2 (3 nM) and GDF5 (30 nM) signaling assays in LLC-PK1 cells transfected with RGM and luciferase-reporter vectors. (*D* and *E*) BMP2 (3 nM) and GDF5 (30 nM) signaling assays in LLC-PK1 cells similar to *B* and *C* but including single amino acid variants of RGMB. Each column in *B*, *D*, and *E* represents an average of data from 32 wells with cells (except *D*, 16 wells). Bars represent SDs. *P* values (Student’s *t* test; two-tailed assuming unequal variance) are shown for selected datasets compared to cells transfected with an empty vector and treated with BMP2/GDF5 (black) or full-length RGMB and treated with BMP2/GDF5 (blue). (*F* and *G*) Cartoon representation of the RGMB_ND_–GDF5 complex. GDF5 protomers are shown in light and dark blue. RGMB_ND_s are shown in light and dark pink. (*G*) Complex rotated 90° relative to *F* along the horizontal axis. Disulfide bonds (orange) are labeled with Roman numerals. The *N*-acetylglucosamine moiety on RGMB Asn120 is shown in gray. (*H* and *I*) RGM–GDF5 interfaces (circled in *F*). Hydrogen bonds between selected atoms (oxygen, red; nitrogen, blue) are shown as dashed gray lines. Water molecules are shown as red spheres in *I*. Distances (Å) between selected atoms are indicated.

Since previous studies revealed a high degree of structural similarity between the GDF5– and BMP2–receptor complexes (8, 26), we wondered whether the BMP2 coreceptor family of the RGMs has a similar effect on GDF5 signaling. We performed a GDF/BMP-responsive luciferase reporter assay in LLC-PK1 kidney cells, which are highly responsive to many BMP/GDF family members and were used to discover that RGMs act as BMP coreceptors (9–11, 17, 21, 27). Here, we show that all full-length membrane-anchored human RGMs enhance BMP2 signaling, consistent with previous observations (Fig. 1 *A* and *B* and *SI Appendix*, Fig. S1 *A* and *B*) (10, 17). When BMP2 was applied at 3 nM (which is below its half-maximal effective concentration, EC_50_), the signaling increased about threefold in cells expressing RGMB and RGMC, and less prominently in cells expressing RGMA. The different levels of BMP2 signal enhancement might be related to different expression levels (*SI Appendix*, Fig. S2 *A* and *B*) and RGM–BMP2 binding affinities (17). RGM constructs lacking the N-terminal domain (RGM_ΔN_) [that was previously shown to be essential for BMP2 binding (17)] failed to enhance BMP2 signaling (Fig. 1*B* and *SI Appendix*, Fig. S1 *A*–*D*), suggesting that modulation of BMP signaling requires RGM–BMP2 binding.

Unexpectedly and in contrast to BMP2 signaling, membrane-anchored RGMs inhibited GDF5 signaling in the same cellular assay. GDF5 signaling was down-regulated about threefold in cells expressing full-length RGMB and twofold for RGMA and RGMC (Fig. 1*C* and *SI Appendix*, Fig. S1 *E* and *F*). This effect was again dependent on the N-terminal domain of RGM, since constructs lacking this domain showed no effect on GDF5 signaling (Fig. 1*C*). Next, we tested whether soluble RGM fragments would also affect signaling. Soluble N-terminal domains of RGMA and RGMB did not attenuate GDF5 signaling as efficiently as membrane-anchored full-length RGMs in LLC-PK1 cells (*SI Appendix*, Fig. S2 *C*–*F*). This may potentially be due to avidity and the requirement for cell surface attachment, where two membrane-bound RGMs are favorably arranged to bind to the GDF5 dimer. The full extracellular domain of RGMB (RGMB_ECD_) inhibited GDF5 signaling similarly to RGMB_ND_, suggesting that the RGMB_ND_, but not the NEO1-binding region of RGMB_ECD_, is essential for the inhibitory function of RGMB (*SI Appendix*, Fig. S2*F*). Intriguingly, when BMP2 and GDF5 were added simultaneously, both the RGM-mediated inhibition of GDF5 signaling and the RGM-mediated activation of BMP2 signaling were diminished (*SI Appendix*, Fig. S2*G*). This suggests that GDF5 can down-regulate RGM-mediated activation of BMP2 signaling, a mechanism that could be essential for a number of biological processes where BMP2 and GDF5 are coexpressed, (e.g., limb and joint development) (28, 29; for recent reviews, see refs. 30 and 31).

Soluble BMPR1A ectodomain protein did not inhibit GDF5 signaling at the concentration used (*SI Appendix*, Fig. S2*H*). However, the fact that neutralizing anti-BMPR1A antibodies down-regulated GDF5 signaling (*SI Appendix*, Fig. S2 *I* and *J*) clearly shows that GDF5 signals are transmitted via BMPR1A into LLC-PK1 cells. Inhibition of the GDF5 signaling by anti-BMPR1A antibodies was not complete and could be further increased by the expression of full-length RGMB (*SI Appendix*, Fig. S2 *I* and *J*). These results suggest that the concentrations of the anti-BMPR1A antibodies (0.5 μM, which relates to a concentration of only about 10- to 25-fold of the equilibrium binding constant measured for the interaction of the respective Fab to soluble BMPR1A protein) were not sufficient to fully abrogate GDF5 signaling. In addition, the anti-BMPR1A antibodies used in this study are monovalent Fab antibody fragments that are much weaker competitors compared to a classic bivalent antibody that can simultaneously bind to two BMPR1A moieties at the cell surface. As would be expected for molecules that bind to an epitope highly overlapping with that for BMP type 1 receptors, soluble RGM fragments could down-regulate GDF5 (as well as BMP2) signals in other cell lines, such as the prechondrogenic (ATDC5) and the mesenchymal (C3H10T1/2) stem cell lines even though the half-maximal inhibitory concentration (IC_50_) varied significantly (*SI Appendix*, Fig. S3).

To further dissect the RGM–GDF5 interactions, we tested three RGMB mutations that impair both RGMB-mediated potentiation of BMP2 signaling and RGMB–BMP2 interactions (*SI Appendix*, Figs. S4 and S5) (17). The two RGMB mutations Gly101Arg and Leu103Glu correspond to RGMC mutations that cause the blood iron overload disease juvenile hemochromatosis (17, 21, 27). The third mutation (RGMB His106Arg) abolished the RGMB–BMP2 interaction (17). In our assay, all three mutations impaired RGMB-mediated potentiation of BMP2 signaling as well as inhibition of GDF5 signaling (Fig. 1 *D* and *E* and *SI Appendix*, Fig. S1 *C*, *D*, *G*, and *H*). This suggests that both the RGMB-inhibitory effect on GDF5 and the RGMB-potentiating effect on BMP2 signaling are mediated by similar interactions between BMP2/GDF5 and membrane-anchored RGMs.

### Crystal Structures of Binary RGM–GDF5 Complexes.

To characterize the RGM–GDF5 interactions, we determined the crystal structure of RGMB_ND_ in complex with GDF5 at 1.7 Å resolution (Fig. 1 *F*–*I* and Table 1). In the complex, the disulfide-linked GDF5 dimer binds two RGMB_ND_ molecules that are related by a noncrystallographic pseudo twofold axis (RMSD of 0.21 Å for 143 equivalent Cα atoms of the 1:1 RGMB:GDF5 complexes) (Fig. 1 *F* and *G*). RGMB_ND_ forms a bundle of three α-helices stabilized by three disulfide bonds (Fig. 1 *F* and *G* and *SI Appendix*, Fig. S5). It binds to the finger region of GDF5 and interacts with both GDF5 molecules of the dimer. The RGMB_ND_–GDF5 interface comprises mixed charged and hydrophobic interactions (containing 116 nonbonded contacts and 5 hydrogen bonds) mediated by 20 residues from each binding partner and buries a total surface area of 1,929 Å^2^ (*SI Appendix*, Fig. S6).

**Table 1.** Data collection and refinement statistics

	RGMA–GDF5	RGMB–GDF5 (crystal form 1)	RGMB–GDF5 (crystal form 2)	RGMC–GDF5	NEO1–RGMB–GDF5
Data collection					
X-ray source	Diamond Light Source	Diamond Light Source	Diamond Light Source	Diamond Light Source	Diamond Light Source
	I04	I03	I04	I03	I04
Space group	P 6_2_ 2 2	P 2_1_	P 6_2_ 2 2	P 6_2_ 2 2	P 6_2_
Unit cell dimensions, *a, b, c* (Å)	97.65, 97.65, 99.82	36.48, 127.75, 39.91	98.86, 98.86, 99.81	98.82, 98.82, 99.23	279.48, 279.48, 142.37
*α*, *β*, *γ* (°)	90.00, 90.00, 120.00	90.00, 99.32, 90.00	90.00, 90.00, 120.00	90.00, 90.00, 120.00	90.00, 90.00, 120.00
Wavelength (Å)	0.9795	0.9762	0.9795	0.9763	0.9795
Resolution (Å)*	49.91**–**2.78 (2.85**–**2.78)	39.38**–**1.65 (1.69**–**1.65)	64.98**–**3.13 (3.21**–**3.13)	85.58**–**2.50 (2.56**–**2.50)	70.19**–**5.50 (5.60**–**5.50)
*R*_meas_ (%)	9.2 (423.8)	10.5 (378.5)	8.3 (330.9)	12.3 (692.7)	27.2 (251.7)
*I*/σ(*I)*	23.4 (0.8)	9.5 (0.5)	23.4 (1.1)	23.9 (0.7)	5.7 (0.8)
Completeness (%)	97.8 (92.0)	99.7 (99.1)	97.6 (95.9)	99.0 (96.7)	99.9 (100.0)
Redundancy	18.5 (19.8)	6.8 (6.3)	35.8 (24.3)	47.8 (36.6)	6.3 (6.5)
*CC* 1/2 (%)	100.0 (59.2)	99.9 (21.2)	100.0 (80.6)	100.0 (44.2)	99.8 (28.1)
Refinement					
Resolution (Å)	48.83**–**2.78	39.38**–**1.65	49.43**–**3.16	85.58**–**2.51	69.87**–**5.50
No. reflections	7,337	42,900	4,299	8,883	20,730
*R*_work_/*R*_free_ (%)	23.4/28.8	19.1/21.9	23.5/26.8	23.8/26.7	32.6/42.8
No. atoms					
Protein	1,265	2,822	1,230	1,230	25,084
Ligand/glycan/ion	13	51	0	0	126
Water	0	142	0	12	0
B factors (Å^2^)					
Protein	163.8	41.3	129.3	108.1	572.8
Ligand/ion	247.0	62.6	–	–	521.6
Water	–	42.0	–	43.1	–
RMSD					
Bond lengths (Å)	0.006	0.006	0.001	0.003	0.012
Bond angles (°)	0.98	1.17	0.38	0.87	1.61
Ramachandran outliers (%)	0.0	0.0	0.0	0.0	0.2
Ramachandran favored (%)	90.4	97.7	95.5	94.7	95.1
MolProbity score	1.5	1.2	1.4	0.9	1.8
MolProbity clash score	2.0	3.6	2.5	0	3.1

* Values in parentheses are for highest-resolution shell.

The RGMB–GDF5 interface is mainly formed by two interaction sites. First, a short linker between helices α2–α3 (Fig. 1*H*) harbors the “RGD-loop” in RGMA and RGMC (“RGN” in RGMB) and interacts with the GDF5 wrist region. The side-chain of RGMB Leu103 inserts into the hydrophobic pocket on GDF5 formed by Trp414, Trp417, Ile449, Tyr490, and Met493 (Fig. 1*H*) and is shielded by a hydrogen bond formed by RGMB Gly101 (“RGD-loop”) and GDF5 Tyr490. Second, the RGMB α3 helix stretches across the two GDF5 fingers. A major interface is formed by the side-chain of RGMB His106 that stacks onto GDF5 Trp417 (Fig. 1*I*). This π**–**π stacking is shielded from the solvent by RGMB Arg100. Our 1.7 Å crystal structure allowed us to identify a network of well-ordered water molecules contributing to the RGMB**–**GDF5 interactions. The side-chain of RGMB His106 forms a hydrogen bond with the amide of Gly101 from the “RGD-loop” and a water molecule that extends a hydrophilic network to Tyr105, Trp414, and Asp416 of GDF5 (Fig. 1*I*). Asp416 is evolutionarily conserved in 12 of 20 GDFs/BMPs and might modulate the specificity of RGM–GDF/BMP interactions (e.g., Asp416 corresponds to Ser343 in BMP9 that does not bind to RGMs) (32) (*SI Appendix*, Fig. S4).

**Fig. 2.**
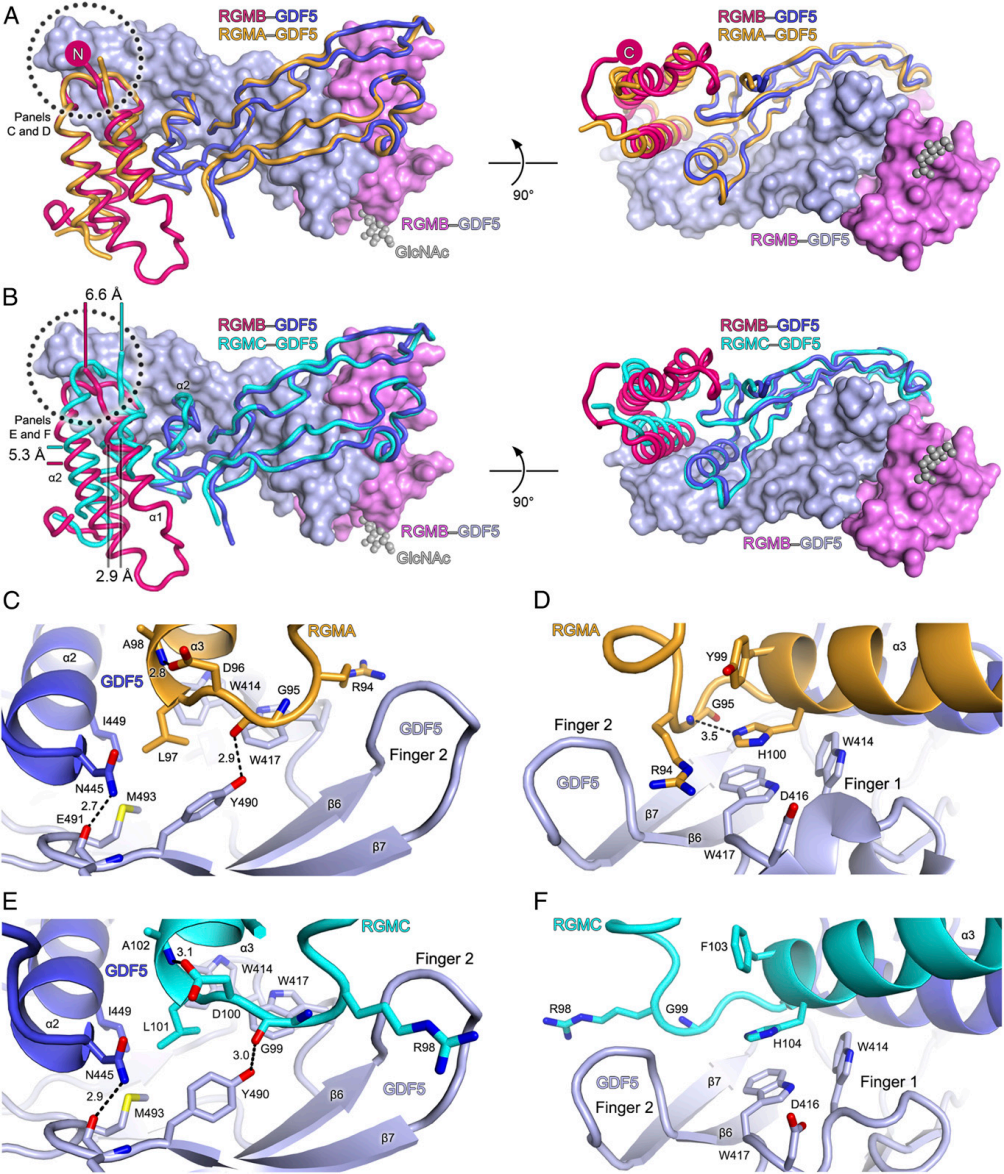
The architecture of human RGM–GDF5 complexes is conserved. (*A*) Superposition of RGMA_ND_–GDF5 (orange) and RGMB_ND_–GDF5 (pink–blue, two RGMB_ND_ and GDF5 molecules are shown as ribbons and two in surface representations) complexes calculated with GDF5 as reference. The two views differ by a 90° rotation around a horizontal axis. The “finger 2” region of GDF5 contacting the RGD/RGN motif of RGMs is circled. (*B*) Superposition of RGMC_ND_–GDF5 (cyan) and RGMB_ND_–GDF5 (pink–blue) complexes. The GDF5 finger 2 contacting the RGD/RGN motif of RGMs is circled. Distances between corresponding Cα atoms of RGMB and RGMC are indicated, highlighting a relative translation of RGMC (compared to RGMA and RGMB) towards the finger 2 of GDF5. (*C*–*F*) Close-up views of the RGM–GDF5 interfaces with molecules colored as in *A* and *B*. Hydrogen bonds between selected atoms are shown as dashed lines. Distances (Å) between selected atoms are indicated in *C*–*E*.

We also determined the structures of the RGMA_ND_–GDF5 and RGMC_ND_–GDF5 complexes (Figs. 1*A* and 2 and Table 1). Superposition of the three RGM–GDF5 complexes reveals common interactions between all three RGMs and GDF5. However, while the GDF5 structure in the three complexes is largely invariant, the relative orientation of the three RGM_ND_ helix bundles towards GDF5 differs (Fig. 2 *A* and *B*). The RGM α3 helix, which is in the core of the RGM–GDF5 interface, undergoes a conformational change. The relative position of the α3 helix differs by ∼5 Å in RGMB_ND_–GDF5 and RGMC_ND_–GDF5 complexes (Fig. 2*B*), with its N terminus being the anchor point for a rotation of RGM_ND_. The pivot point seems to be RGMB Leu103 (RGMA Leu97, RGMC Leu101) (Figs. 1*H* and 2 *C* and *E*). A relative rotation around this leucine permits the side-chain of the preceding arginine (Arg100 in RGMB) to be inserted between GDF5 fingers 1 and 2 in the RGMA– and RGMB–GDF5 complexes (Figs. 1*I* and 2*D*). In the RGMC_ND_–GDF5 complex, however, this arginine side-chain is placed above finger 2 (Fig. 2*F*), potentially explaining the different arrangements of the RGM α3 helix. Taking these data together, we find the linker between RGM α2–α3 helices is essential for the RGM–GDF5 interactions, potentially directing the relative orientation of RGM in the complex. Intriguingly, mutations in the α2–α3 linker of RGMC (Gly99Val, Gly99Arg, Leu101Pro) cause iron overload (21, 27) and impair interactions with the closely related BMP2 (17).

### Interaction Determinants of RGM–GDF5 Complexes.

**Fig. 3.**
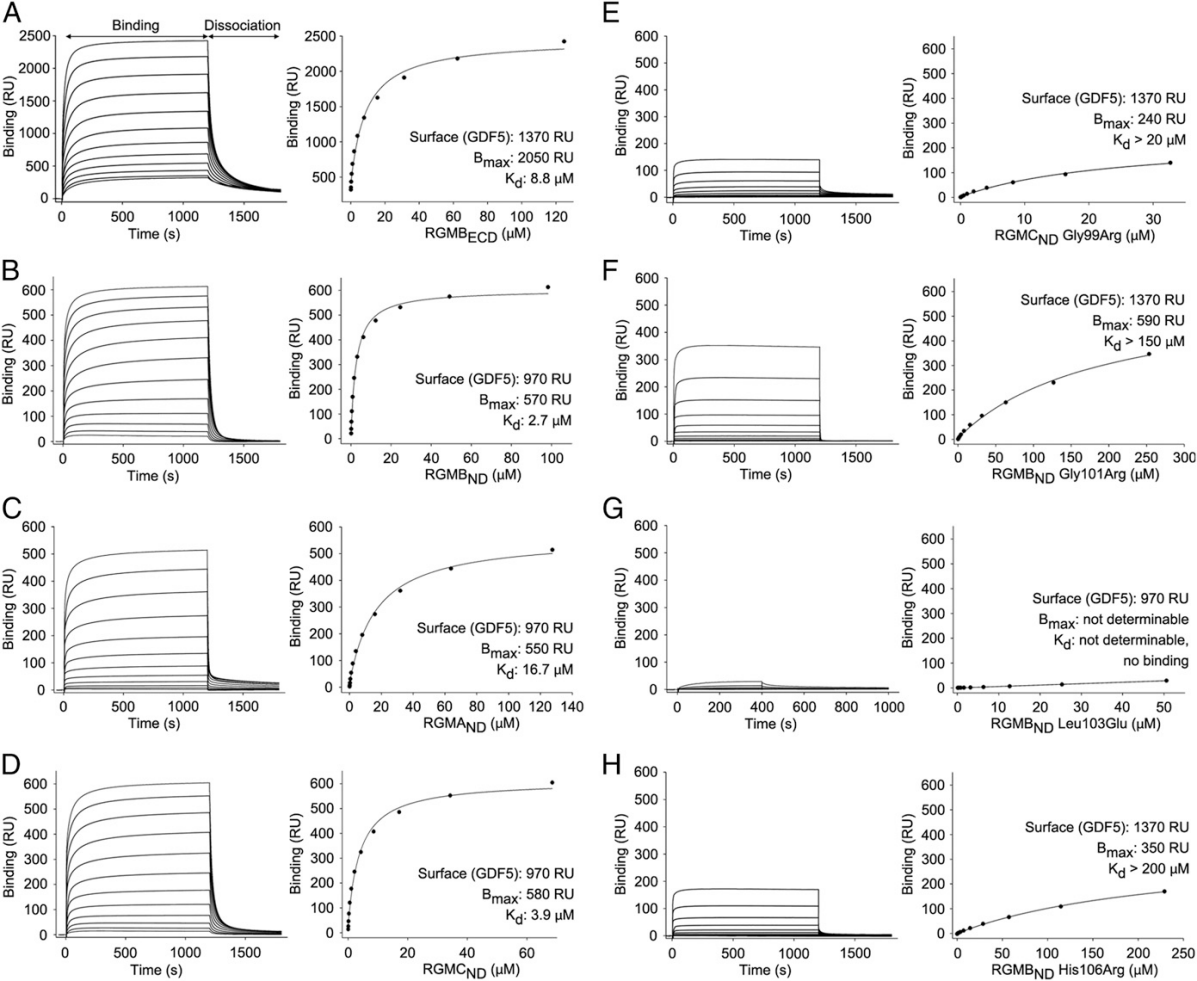
SPR-based equilibrium binding experiments between RGMs and GDF5. (*A*–*H*) SPR-based equilibrium binding experiments showing direct interactions between RGMs and GDF5. The extracellular domain of RGMB (RGMB_ECD_) bound to GDF5 with a similar affinity (*K*_d_ 8.8 μM, *A*) as RGMB_ND_ (*K*_d_ 2.7 μM, *B*), highlighting RGMB_ND_ as the major site mediating RGMB–GDF5 interactions. Mutations of RGM residues at the RGM–GDF5 interface weaken interactions (*E*–*H*). SPR sensorgrams and corresponding isotherms are shown. *B*_max_, maximum response at saturating concentration of analyte (RGM); RU, response units.

We performed surface plasmon resonance (SPR)-based RGM–GDF5 equilibrium binding experiments to dissect the contribution of different RGM domains and residues to GDF5 recognition. GDF5 was immobilized on the SPR chip surface and RGMs were used as analytes. To avoid nonspecific interactions between RGMs and GDF5, which impair the analysis of the dose-dependency of equilibrium binding [as reported for the RGM–BMP2 interaction (17)], a high ionic strength buffer with a 10-fold higher detergent concentration than usually employed was used: 0.5 M NaCl, 20 mM Hepes pH 7.4, 0.05% Tween 20. This elevated detergent concentration generally weakened RGM–BMP2/GDF5 interactions compared to previous studies without altering binding specificity and thus allowed comparative analysis of our RGM mutants (17, 32). Under these conditions the RGMB ectodomain (RGMB_ECD_) bound GDF5 with an apparent affinity (*K*_d_) of 8.8 μM, and the N-terminal domain alone (RGMB_ND_) exhibited a *K*_d_ of 2.7 μM, suggesting that RGMB_ND_ comprises the major domain for GDF5 binding (Fig. 3 *A* and *B*). Under the same conditions, RGMB_ND_ bound to BMP2 with a notably weaker (compared to GDF5) *K*_d_ of 20.1 μM (*SI Appendix*, Fig. S7*A*). GDF5 interacted similarly with RGMC_ND_ (*K*_d_ 3.9 μM) and weaker with RGMA_ND_ (*K*_d_ 16.7 μM) (Fig. 3 *C* and *D*). The juvenile hemochromatosis-causing mutation, Gly99Arg, in RGMC_ND_ and a corresponding mutation, Gly101Arg, in RGMB_ND_ attenuated the interactions with GDF5, *K*_d_ > 20 μM and > 150 μM, respectively (Fig. 3 *E* and *F*). Mutations of RGMB at either of two key RGMB–GDF5 interactions (Leu103Glu and His106Arg) (Fig. 1 *H* and *I*) abolished binding to GDF5 (Fig. 3 *G* and *H*). To compare RGMB–GDF5 interactions to previous BMPR1A–GDF5/BMP2 binding studies (33), we also measured binding under the conditions used in those studies (0.5 M NaCl, 10 mM Hepes pH 7.4, 3.4 mM EDTA, 0.005% Tween 20). Here, binding affinities of RGMB for GDF5 (*K*_d_ 2.7 ± 0.9 nM) and BMP2 (*K*_d_ 4.2 ± 1.2 nM) were similar (*SI Appendix*, Figs. S8 and S9 and Table S1). Finally, we tested the pH dependency of the RGMB–GDF5 interactions using size-exclusion chromatography (SEC) combined with multiangle light scattering (MALS). In solution the 2:2 RGMB–GDF5 complex is stable at pH 7.5, but dissociates at pH 5.5 (*SI Appendix*, Fig. S10), similarly to the RGMB–BMP2 complex.

### Comparison of GDF5/BMP2–Receptor and –RGM Complexes.

**Fig. 4.**
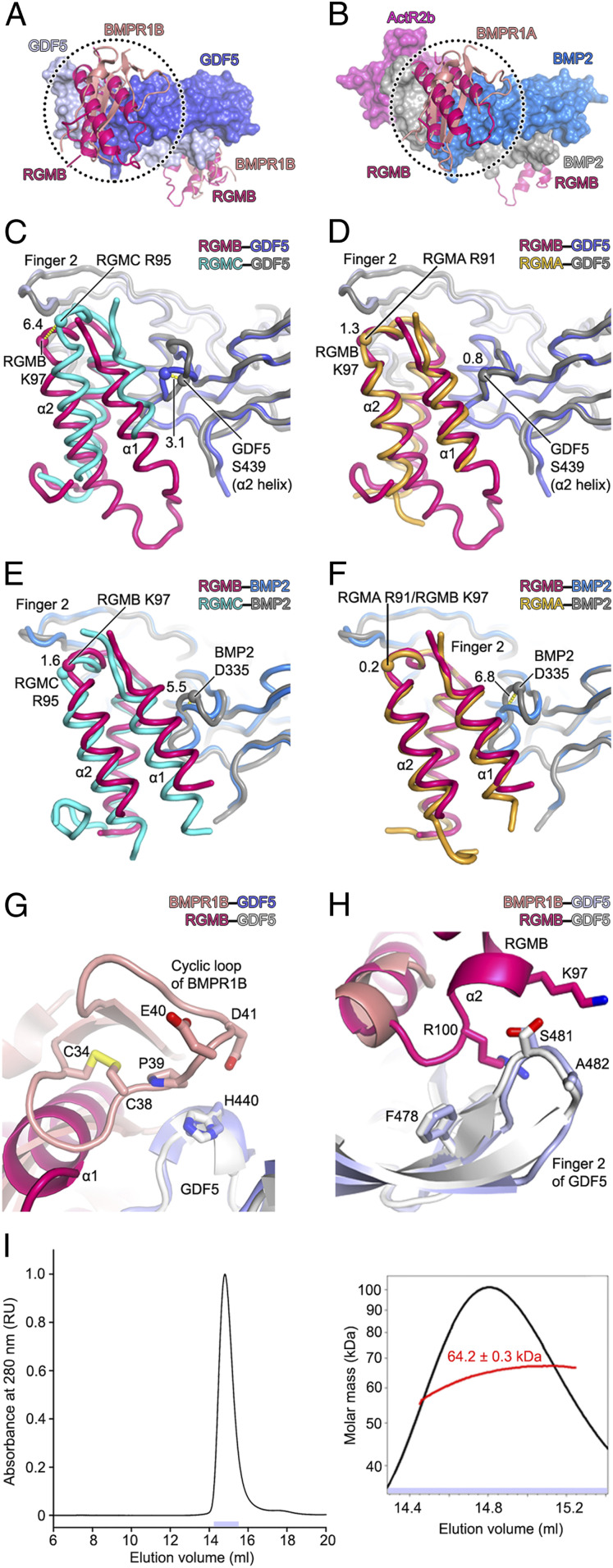
RGMs compete with the type 1 (but not type 2) receptor binding site on BMP2 and GDF5. (*A*) Superposition of BMPR1B–GDF5 (GDF5 and BMPR1B are shown in surface and cartoon representations, respectively; PDB ID code 3EVS) and RGMB_ND_–GDF5 (only RGMB is shown). (*B*) Superposition of BMPR1A–BMP2–ActR2b (shown in surface representation, except BMPR1A shown as a cartoon; PDB ID code 2H62) and RGMB_ND_–BMP2 (only RGMB is shown; PDB ID code 4UHZ). (*C* and *D*) Superposition of RGMB_ND_–GDF5 and RGMC_ND_–GDF5 (*C*), and RGMB_ND_–GDF5 and RGMA_ND_–GDF5 (*D*) complexes. (*E* and *F*) Superposition of RGMB_ND_–BMP2 (PDB ID code 4UI0) and RGMC_ND_–BMP2 (PDB ID code 4UI1) (*E*), and RGMB_ND_–BMP2 and RGMA_ND_–BMP2 (*F*) complexes. Distances (Å) between selected Cα atoms (shown as spheres) are indicated with dashed lines. (*G* and *H*) Superposition of BMPR1B–GDF5 (PDB ID code 3EVS) and RGMB_ND_–GDF5 reveals regions of GDF5 that contact BMPR1B (but not RGMB) (*G*) and regions of GDF5 that contact RGMB (but not BMPR1B) (*H*). (*I*) SEC-MALS analysis of the RGMB_ND_–GDF5–ActR2b complex. The experimental molecular mass of the RGMB_ND_:GDF5:ActR2b complex is 64.2 kDa, corresponding to a 2:2:2 arrangement (theoretical molecular mass: 71.2 kDa). Traces of absorbance at 280 nm are shown as continuous black lines. The *Inset* shows a close-up of the peak with indicated molecular weight values (with associated statistical uncertainties, calculated using the Astra software from Wyatt Technologies).

Superposition of the BMPR1B–GDF5 and BMPR1A–BMP2–ActR2b complexes with binary RGM–GDF5 and RGM–BMP2 complexes, respectively, reveals that RGMs and type 1 receptors (BMPR1A and BMPR1B) bind to highly overlapping epitopes on GDF5 and BMP2 (Fig. 4 *A* and *B*). However, in contrast to previously determined RGM–BMP2 complexes (17), the relative orientations of our three RGM_ND_s bound to GDF5 vary (Fig. 4 *C*–*F*). Strikingly, the superposition of GDF5-bound RGMB_ND_ and RGMC_ND_ requires translation by 5.6 Å and rotation by 15.4° (Fig. 4*C*). For comparison, the superposition of BMP2-bound RGMB_ND_ (two crystal forms) and RGMC_ND_ requires translation by 3.0 to 3.6 Å and rotation by 6.9 to 8.2° (Fig. 4*E*). While RGMs bind to at least five different BMPs (32), differences in their interactions might lead to distinct biological effects.

Our structures also reveal GDF5 regions engaged specifically in either BMPR1B or RGM recognition (Fig. 4 *G* and *H*). GDF5 His440 interacts with a cyclic loop of BMPR1B (Fig. 4*G*) (but not with any RGMs), whereas a tip of finger 2 (Phe478–Ser481) of GDF5 interacts with C-terminal residues of the RGM α2 helix (Fig. 4*H*). GDF5–type 2 receptor interactions are mediated by the knuckle epitope in finger 2, which is not part of the RGM–GDF5 interface. Nevertheless, RGMs were found to modulate interactions between BMP2/4 and their type 2 receptors (34) although it is unclear whether these receptors can form ternary complexes with RGMs–BMPs/GDFs. Using SEC-MALS, we show that RGMB_ND_, GDF5 and ActR2b form a ternary complex in solution (Fig. 4*I*). This suggests that RGMs might modulate BMP/GDF–BMP type 2 receptor interactions (34) by altering the conformation of finger 2 and the knuckle epitope (Fig. 4*H*).

Next, to understand the opposing role of RGM on GDF5 and BMP2 signaling, we tested the effect of RGMB on signaling by two GDF5 mutants (GDF5 Arg438Leu and GDF5 Arg438Ala) that bind to BMPR1A with higher affinity, similar to that of BMP2, thus mimicking a BMP2-like behavior for BMPR1A binding (26) (*SI Appendix*, Table S1). Both GDF5 mutants activated signaling as found for wild-type GDF5 in LLC-PK1 cells but with a dose-dependency similar to BMP2 as would be expected for a BMP2 mimic (*SI Appendix*, Fig. S2 *K* and *L*). However, signaling by both GDF5 mutants was still inhibited by RGMB similar to that found for wild-type GDF5. Using an SPR-based binding assay we could furthermore show that these GDF5 mutants bind to all RGM ectodomains (RGMA_ECD_, RGMB_ECD_, and RGMC_ECD_) with affinities identical to wild-type GDF5, indicating that only BMPR1A binding is changed by these mutations (*SI Appendix*, Fig. S9). Our additional analyses using these GDF5 mutants clearly indicate that the opposing effect of RGM on BMP2/GDF5 signaling cannot be explained with a simple RGM/type 1 receptor competition mechanism as this would have been addressed with these GDF5 mutants. Hence, an additional far more complex mechanism must play a role that allows recognition and discrimination of BMP2 and GDF5, despite structure analyses showing seemingly identical interaction schemes for both BMP ligands.

### RGMB Bridges NEO1 and GDF5 in a Ternary Inhibitory Complex.

**Fig. 5.**
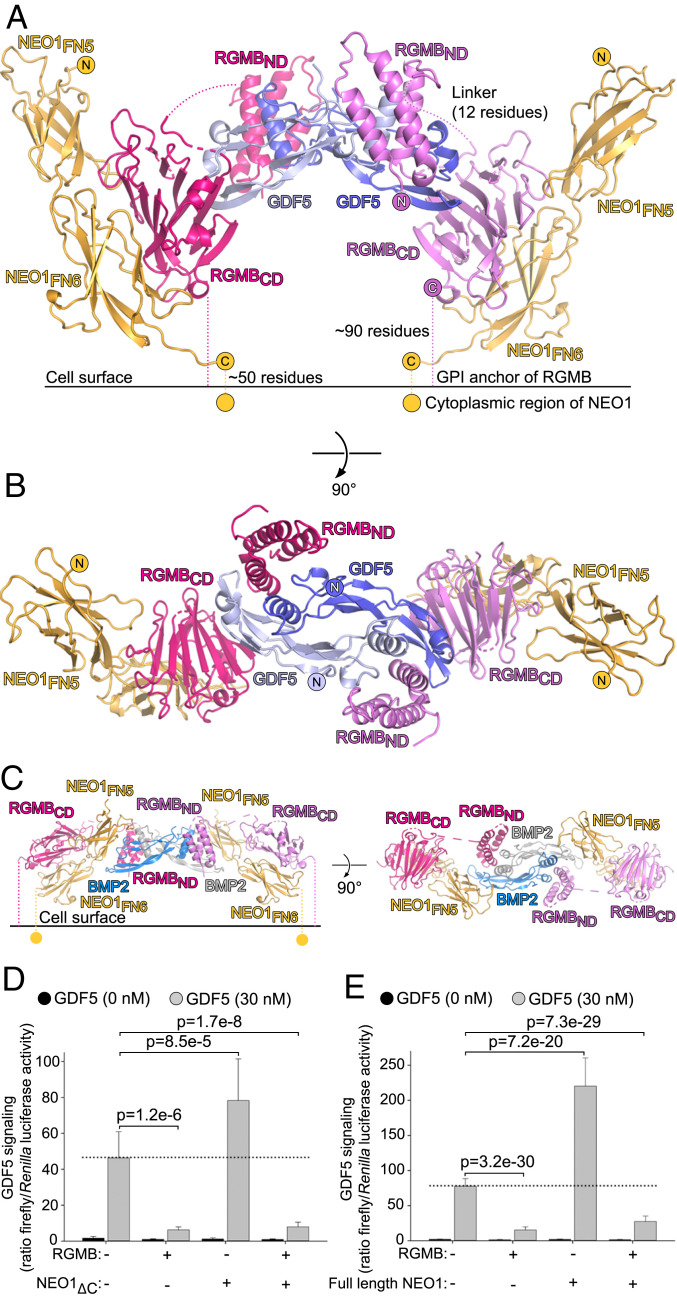
Structure of the NEO1–RGMB–GDF5 complex. (*A* and *B*) Ribbon representation of the NEO1–RGMB–GDF5 complex. RGMB and GDF5 are colored as in Fig. 1, NEO1 is in yellow. The RGMB_ND_–RGMB_CD_ linker and C termini of RGMB are shown as dotted lines. View in *A* (parallel to cell membrane) and *B* (view from the top) differ by a 90° rotation around a horizontal axis. N and C termini are marked. (*C*) The NEO1–RGMB–BMP2 complex (17) shown in similar orientations as the NEO1–RGMB–GDF5 assembly in *A* and *B*. (*D* and *E*) GDF5 (30 nM) signaling is inhibited by RGMs in the presence or absence of NEO1 (lacking the cytoplasmic domain, NEO1_ΔC_), *D*; full-length NEO1, *E* in LLC-PK1 cells. Each column represents an average of data from 16 (*D*) or 31 to 32 (*E*) wells with cells. Bars represent SDs. *P* values (Student’s *t* test; two-tailed assuming unequal variance) are shown for selected data sets compared to cells transfected with an empty vector and treated with GDF5.

How the NEO1 and GDF5/BMP signaling pathways, which both control chondrogenesis and iron homeostasis, are interfacing, is only poorly understood (4, 11, 18, 22–24, 26). We hypothesized that GDF5 might form a ternary complex with RGMB and NEO1 similar to the ternary RGMB–BMP2–NEO1 assembly (17). To test this hypothesis, we determined the structure of the ternary complex between the two membrane proximal NEO1 fibronectin-type III domains (NEO1_FN56_) (16), RGMB_ECD_ and GDF5 to 5.5 Å resolution. In the complex, the GDF5 dimer binds to two RGMB ectodomains in a similar arrangement to that in the binary RGMB_ND_–GDF5 complex (Fig. 5 *A* and *B* and *SI Appendix*, Fig. S11). RGMB_ND_ is connected to the RGMB C-terminal domain (RGMB_CD_) via a flexible linker (∼12 residues), as suggested by its fragmentary electron density. RGMB_CD_ is sandwiched between GDF5 and NEO1. A hydrophobic face of RGMB_CD_ (Phe162–Phe166, Arg172, Tyr268–Glu270, Phe281, Ala292) interacts with a hydrophobic region of GDF5 (Ile419–Leu422, Val485–Tyr487, Leu477–Ile479). However, this interaction is likely to play a minor role in the RGMB–GDF5 interactions, as RGMB_ND_ is sufficient to bind to GDF5 with high affinity (Fig. 3 *A* and *B*). RGMB_CD_ uses an opposite face of the β-sandwich to bind NEO1, resembling the main interface previously observed in the binary NEO1–RGMB complex (16).

The 2:2:2 NEO1–RGMB–GDF5 architecture is compatible with a potential arrangement of RGMs, NEO1, and GDF5 on the cell surface (Fig. 5 *A* and *B*). C termini of RGMB and NEO1 point in the same direction and could be accommodated on the surface of one cell to control downstream signaling. This overall arrangement resembles the previously determined NEO1–RGMB–BMP2 structure (17) (Fig. 5*C*), since the RGMB_ND_–BMP2/GDF5 and RGMB_CD_–NEO1 interfaces are conserved, and the C termini of RGMB and NEO1 point in the same direction so that both can be accommodated on the surface of one cell. Both NEO1–RGMB–GDF5/BMP2 complexes demonstrate the modular architecture of RGMs where RGM_ND_ and RGM_CD_ can control BMP/GDF and NEO1 signaling, respectively. This raises the question of whether NEO1 affects GDF5 signaling and the inhibitory effect of RGMs on GDF5. To address this, we performed GDF5-responsive cellular signaling assays in LLC-PK1 cells expressing either full-length NEO1 (NEO1_FL_) or a NEO1 construct replacing its cytoplasmic domain with a green fluorescent protein (NEO1_ΔC_) (17). In both cases, RGMB could inhibit GDF5 signaling at similar levels compared to RGMB alone (Fig. 5 *D* and *E*). Interestingly, both NEO1_ΔC_ and NEO1_FL_ increased GDF5 signaling in the absence of RGMB (Fig. 5 *D* and *E*), a similar effect to that previously shown for BMP2 signaling in chondrocytes (23) and myoblasts (35). However, the molecular basis of NEO1-mediated enhancement of BMP2/GDF5 signaling remains unknown. In summary, our structural analysis of the ternary NEO1–RGMB–GDF5 complex combined with cellular assays reveal the modular architecture of RGMB where its N-terminal domain acts as a robust inhibitor of GDF5 signaling, whereas its C-terminal domain interacts with NEO1.

## Discussion

A comparison of RGM–BMP2 structures (17) with the RGM–GDF5 structures presented here not only identified common principles of the RGM–BMP/GDF interaction but also showed structural differences. These additional data will facilitate establishing a recognition mechanism for the RGM–BMP/GDF interplay that can explain why certain BMPs, such as BMP9, cannot bind RGMs (32). As a common element, our RGM–BMP/GDF structures suggest that RGMs could principally compete with BMP/GDF type 1 receptors for binding of the ligands (Fig. 4 *A* and *B*). Consistently, membrane-anchored (and dependent on the cell type, also soluble) RGMs inhibited GDF5 signaling. However, paradoxically, membrane-anchored RGMs enhance BMP2 signaling, although BMP2 and GDF5 share a high sequence conservation (56% in the signaling domain) (*SI Appendix*, Fig. S4) and structural similarity between their complexes with RGMs (Fig. 4 *A*–*F*).

**Fig. 6.**
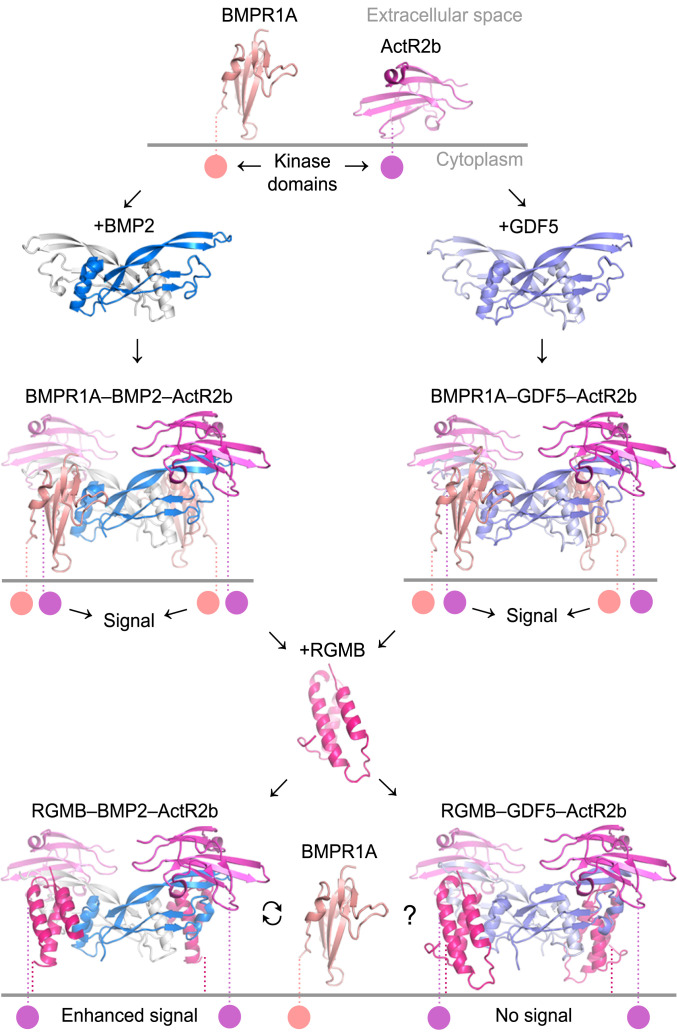
Control of BMP2 and GDF5 signaling by RGMs. Dimeric BMP2 and GDF5 ligands assemble a complex comprising type 1 and type 2 receptors (BMPR1A and ActR2b, respectively) to activate downstream signaling. Type 1 receptors and RGMs occupy a highly overlapping epitope on BMP2 and GDF5, However, while membrane-anchored RGMs enhance BMP2 signaling, GDF5 signaling is inhibited by RGMs. Ternary signaling complexes were modeled based on BMPR1A–BMP2–ActR2b (PDB ID code 2H62), BMPR1A–GDF5 (PDB ID code 3QB4) and RGMB–GDF5 (present study). The C-terminal domain of RGM is not shown for simplicity.

A very simple explanation for this contradictory effect of RGMs could be the different binding affinities of GDF5 and BMP2 to BMPR1A and RGMB. BMP2 binds tightly to BMPRIA (*K*_d_ 59 ± 29 nM) (see also ref. 33) and with only 10-fold higher affinity to RGMB (*K*_d_ 4.2 ± 1.2 nM). Thus, BMPR1A might just displace RGMB from the membrane-anchored RGMB–BMP2 complexes, thereby triggering signaling. In this working model, RGMB would promote BMP2 signaling by increasing the local BMP2 concentration at the cell. In contrast to BMP2, GDF5 also binds with high affinity to RGMB (*K*_d_ 2.7 ± 0.9 nM) but interacts more weakly with BMPR1A (*K*_d_ 125 ± 3 nM). In the case of GDF5, BMPR1A might be unable to effectively compete with RGMB, leaving GDF5 sequestered in an inactive assembly on the cell (Fig. 6). However, when we tested this working hypothesis we found that GDF5 mutants that exhibit BMP2’s high affinity for BMPR1A were still inhibited by membrane-anchored RGMB similarly to wild-type GDF5. This suggests that the differential RGM-mediated modulation must occur via a more complex mechanism potentially involving unknown cofactors and/or endocytosis mechanisms.

An alternative model for RGM-mediated function on BMPs was proposed by Healey et al. (17). The authors could only observe BMP2 potentiation of membrane-attached RGMB and no effect of soluble RGMs in LLC-PK1 cells. Healey et al. proposed a model whereby the RGM–BMP2 complex (potentially containing the type 2 receptor) may be shuttled to endosomes, dissociate at lower pH (4.5 to 6.5), form a ternary type 1–BMP2–type 2 receptor complex in the endosome and thereby activate signaling proximal to the nucleus. Importantly, in this report we show that a type 2 receptor forms a ternary complex with GDF5 and RGMB at pH 7.4 (Fig. 4*I*). We also show that the RGMB–GDF5 complex exists in solution at pH 7.5 but dissociates at pH 5.5 (*SI Appendix*, Fig. S10), suggesting that a signaling model based on endocytosis may also be valid for GDF5. Similar signaling models have been suggested for the TGF-β family member NODAL and its receptor interactions (36), as well as for the BMP9/10-specific coreceptor Endoglin, which competes with BMP-binding site of type 2 receptors (37, 38). Additional experiments are needed to uncover how RGMs function at a mechanistic level. Intriguingly, if GDF5 and BMP2 are present simultaneously, the RGM-mediated effect is neutralized. It would be exciting to relate our molecular and cellular analysis to in vivo studies in tissues where RGMs, NEO1, and GDF5 are coexpressed (*SI Appendix*, Fig. S12).

The ternary 2:2:2 arrangement observed in our NEO1–RGMB–GDF5 (Fig. 5 *A* and *B*) and the previously reported NEO1–RGMB–BMP2 (17) complexes, combined with the active signaling conformation of the 2:2 complex between NEO1 and the C-terminal domain of RGMB (16), suggest a mode of clustering in which RGM bridges the dimers of BMP/GDF and NEO1 at the cell surface. Such a mechanism could potentially control the subcellular localization of the components, as suggested in the developing growth cone, where inhibition of BMP/GDF signaling blocks recruitment of NEO1 into lipid rafts in an RGMA-dependent fashion (39), or in chondrocytes, where NEO1 regulates BMP receptor association with lipid rafts, a process that is dependent on both the BMP/GDF ligand and RGMs (23). Moreover, it is unknown how NEO1 expressed in hepatocytes inhibits secretion of RGMC, which is released by proteolytic cleavage and cleavage of its GPI anchor (18, 40). The ternary NEO1–RGMB–BMP2/GDF5 complexes show how NEO1 could sequester secreted RGMC. The absence of NEO1 in NEO1-deficient mice could lead to a diffusion of secreted RGMC, inhibition of BMP signaling in the liver and, thus, iron overload (18, 22).

RGMs have been implicated in an increasing number of diseases, ranging from cancer and multiple sclerosis to iron overload disorders. An interesting observation in the central nervous system is the consistent up-regulation of RGMs following a variety of traumas (e.g., neurodegeneration or physical trauma). Blocking RGMA with antibodies is moving into the spotlight of translational studies (41) and clinical trials of RGMA-blocking antibodies in multiple sclerosis patients are ongoing (ClinicalTrials.gov NCT02601885). Similarly, an inhibitory anti-RGMC antibody was shown to reverse anemia caused by high Hepcidin level (up-regulated by BMP6 signaling) in mammals (42–44). Interestingly, both anti-RGMA and -RGMC antibodies bind to the RGM_ND_, which interacts with GDF5 and BMP2, the latter of which were indicated as neurotrophic factors and are being investigated for possible treatment in neurodegenerative diseases, such as Parkinson’s disease (45, 46; for review, see ref. 47). This raises the question of whether neuron regeneration or correction of anemia is primarily mediated via the NEO1 or BMP/GDF signaling pathway. Our structures reveal specific regions on RGM_ND_ (RGMA Leu97, RGMC Leu101) that could be blocked to inhibit the BMP/GDF pathway but preserve NEO1 signaling. Similarly, the NEO1–RGMB–BMP2/GDF5 complexes point towards specific residues of RGM_CD_ as targets for therapeutics that could block NEO1–RGM interactions but preserve the RGM–BMP/GDF signaling. Our results provide a structural basis for the assessment of therapeutic anti-RGM molecules in terms of their ability to specifically preserve or block either the NEO1 or the RGM–BMP/GDF signaling pathways with consequent benefits and risks.

## Materials and Methods

### Production of RGMs, BMPR1A, and NEO1.

Human RGMA_ND_ (residues Ser46**–**Ser139, UniProtKB Q96B86-1), RGMB_ND_ (Gln53**–**His136, Q6NW40-1), RGMB_ECD_ (Gln53**–**Ser411, Q6NW40-1), RGMC_ND_ (Gln36**–**Pro145, Q6ZVN8-1), BMPR1A_ECD_ (Thr55**–**Val141, P36894-1) (17), fibronectin type-3 domains 5 and 6 of mouse NEO1 (Thr883**–**Asn1123, P97798-4) (16) were cloned into the pHLsec vector (48), resulting in an expression construct with an N-terminal secretion signal, followed by Glu-Thr-Gly, the target protein and a C-terminal Gly-Thr-Lys-His_6_ tag. Proteins for crystallization were expressed by transient transfection in HEK-293T cells (ATCC CRL-11268) in the presence of the class I α-mannosidase inhibitor kifunensine for ∼3 to 5 d at 37 °C (48). Media with secreted proteins were centrifuged (7,000 × *g*, 1 h, 18 °C), filtered (0.22-μm polyethersulfone membrane; Millipore), and dialyzed against 200 mM NaCl, 20 mM Tris⋅HCl pH 8.0 using a QuixStand benchtop diafiltration system (GE Healthcare) (∼21 °C, ∼3 to 6 h). Proteins were purified using immobilized metal (cobalt) affinity chromatography (IMAC, TALON resin; Clontech). For crystallization, RGMs and NEO1 were deglycosylated with endoglycosidase F1 (∼10 μg per mg of target protein, ∼2 to 12 h, 21 °C) to cut the Asn-linked glycans down to one *N*-acetylglucosamine moiety, concentrated to ∼1 to 10 mg mL^−1^ and further purified by SEC (typically in 150 mM NaCl, 10 mM Hepes pH 7.5) before complex formation. RGM_ND_ constructs and BMPR1A_ECD_ for cellular and binding assays were produced without kifunensine and kept fully glycosylated. RGM_ND_ constructs and BMPR1A_ECD_ used for cellular assays were dialyzed against 37 mM NaCl, 2.7 mM KCl, and 10 mM phosphate buffer (pH 7.4 at 25 °C) before use. Wild-type GDF5 and GDF5 variants Arg438Ala and Arg438Leu were produced in *Escherichia coli*, refolded, and purified as described previously (7). Soluble RGM extracellular domains for SPR-based binding studies between RGMs and GDF5 mutants were produced in FreeStyle 293 cells by transient expression. Expression levels of RGMs in HEK293 cells were analyzed using primary anti–His-tag antibody followed by a secondary antibody coupled to peroxidase and chemiluminescence (see *SI Appendix* for details).

### Formation and Crystallization of RGM-Containing Complexes.

RGM_ND_–GDF5 and NEO1–RGMB_ECD_–GDF5 complexes were crystallized using sitting-drop vapor diffusion with 100-nL protein solution plus 100-nL reservoir solution per droplet in 96-well Greiner plates at 21 °C (49). Purified RGMA_ND_ and GDF5 were mixed (1:1 mol:mol; ∼1.5 mg mL^−1^) in 100 mM NaCl, 27 mM Hepes pH ∼7.5, 170 mM NDSB-256, incubated for ∼12 h at 4 °C and concentrated (Amicon Ultra-4 centrifugal filters, 3-kDa molecular mass cutoff) to 7.8 mg mL^−1^ and crystallized in 0.2 M ammonium acetate, 0.1 M sodium citrate tribasic dihydrate pH 5.5, 24% (vol/vol) polyethylene glycol (PEG) 400. Crystals were cryoprotected in reservoir solution supplemented with 10% (vol/vol) PEG 400 before transferring into liquid nitrogen. For RGMB_ND_–GDF5 (crystal form 1), RGMB_ND_, ActR2b, and GDF5 were mixed (3.2:2.4:1 mol:mol:mol, ∼1.3 mg mL^−1^) in 0.9 M NaCl, 20 mM Hepes pH 7.4, incubated for ∼12 h at 4 °C, followed by SEC in 0.5 M NaCl, 20 mM Hepes pH 7.4 (HiLoad 16/60 Superdex column; GE Healthcare, 21 °C). SEC fractions containing a ternary complex were concentrated to 5.3 mg mL^−1^ and crystallized in 0.2 M Li_2_SO_4_, 0.1 M Hepes pH 7.5, 25% (vol/vol) PEG 3350. Crystals were cryoprotected in reservoir solution supplemented with 30% (vol/vol) glycerol. For RGMB_ND_–GDF5 (crystal form 2), RGMB_ND_ was deglycosylated after IMAC for ∼2 h at 21 °C in 150 mM NaCl, 10 mM Hepes pH 7.5, then methylated following the described standard procedure in Walter et al. (50) and purified by SEC in 150 mM NaCl, 10 mM Tris⋅HCl pH 8.0. Purified RGMB_ND_ (methylated) and GDF5 were mixed (1:1 mol:mol; 1.6 mg mL^−1^) in 130 mM NaCl, 15 mM Tris⋅HCl pH 8.0, 130 mM NDSB-256, incubated for ∼12 h at 4 °C, concentrated to 8.2 mg mL^−1^ and crystallized in 0.2 M (NH_4_)_2_SO_4_, 0.1 M sodium acetate pH 4.6, 35% (wt/vol) pentaerythritol ethoxylate (15/4 EO/OH; average molecular mass 797 Da). Crystals were cryoprotected in reservoir solution supplemented with 30% (vol/vol) glycerol. Purified RGMC_ND_ and GDF5 were mixed (1:1 mol:mol; ∼1 mg mL^−1^) in 60 mM NaCl, 30 mM Hepes pH ∼7.5, 0.2 M NDSB-256, incubated for ∼12 h at 4 °C, concentrated to 6.9 mg mL^−1^ and crystallized in 1 M LiCl, 0.1 M citric acid pH 4.0, 8% γ-butyrolactone. Crystals were cryoprotected in reservoir solution supplemented with 3.5 M LiCl_2_. For NEO1**–**RGMB_ECD_**–**GDF5 crystallization, the NEO1**–**RGMB_ECD_ complex was formed first. NEO1 and RGMB_ECD_ were mixed (1.2:1 mol:mol; ∼1.3 mg mL^−1^ in 150 mM NaCl, 10 mM Hepes pH 7.5), incubated for ∼12 h at 4 °C and purified using SEC (150 mM NaCl, 10 mM Hepes pH 7.5; HiLoad 16/60 Superdex 200 column, GE Healthcare, 21 °C). Purified NEO1**–**RGMB_ECD_ complex was mixed with GDF5 (1:1.5, mol:mol) in 90 mM NaCl, 21 mM Hepes pH 7.5, 39 mM NDSB-256, incubated for ∼3 h at 4 °C, and purified using SEC (150 mM NaCl, 10 mM Hepes pH 7.5; HiLoad 16/60 Superdex 200 column, GE Healthcare, 21 °C). SEC fractions containing NEO1**–**RGMB_ECD_**–**GDF5 were concentrated to 8.0 mg mL^−1^ and crystallized in 0.1 M NaCl, 20 mM MES pH 6.7, 6.6% (wt/vol) PEG 4000. For data collection, crystals were cryoprotected at 100 K in reservoir solution supplemented with 25% (vol/vol) glycerol.

### Data Collection, Structure Determination, and Refinement of RGM-Containing Complexes.

Data were collected at the Diamond Light Source (United Kingdom), indexed, and integrated using XDS (51), scaled using AIMLESS (52) in xia2 (53). RGM_ND_–GDF5 structures were solved by molecular replacement using Phaser (54) and RGM_ND_s from RGM_ND_–BMP2 complexes [PDB ID codes 4UHY, 4UI0, and 4UI1 for RGMA, RGMB, and RGMC, respectively (17)] and GDF5 [1WAQ (7)] structures as search models. Diffraction data from RGMB_ND_–GDF5 (crystal form 2) and RGMC_ND_–GDF5 crystals were anisotropic. Thus, scaled and merged data were anisotropy-corrected with the STARANISO webserver (Global Phasing) prior to refinement. Estimated diffraction limits of ellipsoid fitted to diffraction cutoff surface for RGMB_ND_–GDF5 (crystal form 2): 3.29 Å, 3.29 Å, 3.0 Å in 0.894a*-0.447b*, b*, and c* directions, respectively; for RGMC_ND_–GDF5: 2.73 Å, 2.73 Å, 2.35 Å in 0.894a*-0.447b*, b*, and c* directions, respectively. All structures were refined using Coot (55) and Phenix (56). The ternary NEO1–RGMB_ECD_–GDF5 complex was solved by molecular replacement using complexes of the RGMB_ND_–GDF5 (crystal form 1) and NEO1–RGMB_CD_ [PDB ID code 4BQ6 (16)] as search models. RGMB_ND_–GDF5 and NEO1–RGMB_CD_ complexes were refined as rigid bodies (9 in total) with one translation, libration, screw (TLS) group per protein domain (30 in total) in Phenix. Nine rigid bodies comprised three RGMB_ND_–GDF5 complexes (rigid body 1: Chains A, B, C, D; 2: Chains G, H, I, J; 3: Chains M, N, O, P) and six NEO1–RGMB_CD_ complexes (rigid body 4: Chains c and E; 5: Chains d and F; 6: chains i and K; 7: Chains j and L; 8: Chains o and Q; 9: Chains p and R) (*SI Appendix*, Fig. S11). Refinement of the NEO1–RGMB_ECD_–GDF5 complex using single chains as rigid bodies (21 rigid bodies in total; disulfide linked chains A/B, G/H and M/N of GDF5 were treated as 3 distinct rigid bodies) gave a model with minor clashes (e.g., between chains j and L that appear less ordered in the crystal) (*SI Appendix*, Fig. S11) as quantified by increase of MolProbity all-atom clash score from 3.1 to 7.3 (57). Thus, the ternary NEO1–RGMB_ECD_–GDF5 complex was refined using nine rigid bodies described above. The quality of structures was assessed using Coot (55) and MolProbity (57). Figures of structures were prepared using PyMOL (The PyMOL Molecular Graphics System, v2.0, Schrödinger).

### SPR-Based Binding Studies.

Initial SPR experiments were performed using a ProteOn XPR36 instrument (Bio-Rad). All measurements were performed at 25 °C and a flowrate of 100 to 200 µL min^−1^ using 10 mM Hepes pH 7.4, 500 mM NaCl, 3.4 mM EDTA, 0.005% (vol/vol) Tween20 (HBST_500_). BMP/TGF-β ligands were immobilized onto GLC sensor chips (Bio-Rad) by primary amino coupling after EDAC/sulfo-NHS activation following the manufacturer’s recommendation. For SPR data acquisition, recombinant full-length mature RGM proteins lacking the C-terminal residue, which is modified to carry the membrane-anchoring glycolipid moiety, were perfused as analyte over the biosensor usually employing six different concentrations. Association was observed for 120 to 240 s, dissociation was initiated by perfusing HBST_500_ buffer and monitored for 40 to 200 s. Biosensor regeneration was performed by a 60-s injection of 100 mM glycine pH 1.5 and then 4 M MgCl_2_ (flow rate during regeneration 30 µL min^−1^). The association and dissociation phase of the sensorgrams were fitted with a Langmuir-type 1:1 interaction model using the software ProteOn Manager v3.1 (Bio-Rad). Data were only interpreted if χ^2^ values were ≤10% of the maximal signal amplitude (RU_max_). Equilibrium binding constants *K*_d_ were calculated from the equation *K*_d_ = *k*_off_/*k*_on_. All affinities are termed “apparent” *K*_d_s, indicating that the absolute values might differ from values obtained by other interaction analysis methods. Differences in affinities and rate constants determined by this set-up greater than twofold were considered significant.

Sensorgrams of RGMB–BMP2/GDF5 interactions derived with the initial set-up were difficult to analyze due to biphasic binding hinting towards nonspecific binding and usually required all fitting parameters to be treated local instead employing global fitting routines. Hence, additional SPR experiments were performed using a different set-up and employing a Biacore T200 instrument (GE Healthcare) at 25 °C. GDF5 and BMP2 were covalently linked to the surface of a CM5 chip (Biacore) via primary amine coupling. A relatively high ionic strength running buffer with detergent (0.5 M NaCl, 20 mM Hepes pH 7.4, 0.05% Tween 20; flow rate 15 µL min^−1^) was used to minimize any nonspecific protein–protein and protein–chip surface interactions. Site-directed mutagenesis of RGMs was performed by a two-step overlap extension PCR and constructs were verified by DNA sequencing. All RGM constructs were purified by SEC in SPR running buffer before use, and a 1:2 dilution series was prepared. After each measurement, the chip surface was regenerated with 4 M MgCl_2_ (100 μL min^−1^, 2 min). The signal from experimental flow cells was corrected by subtraction of a buffer and reference signal from a flow cell without coupled protein. Equilibrium dissociation constants (*K*_d_s) were calculated using BIAevaluation 3.0 program (Biacore) and a 1:1 Langmuir binding isotherm model: *y* = (*B*_max_ × *x*)/(*K*_d_ + *x*); where *y* is binding response, *x* is the analyte concentration, and *B*_max_ is the maximum analyte binding. Analyte concentrations were determined from the absorbance at 280 nm using a NanoDrop ND-1000 spectrophotometer (Thermo Fisher Scientific) and the calculated molar extinction coefficients from ProtParam webserver.

### Cellular Assays of BMP2 and GDF5 Signaling in LLC-PK1 Cells.

LLC-PK1 pig kidney cells (ATCC CL-101) were grown in DMEM high-glucose media (D6546, Sigma) supplemented with 2 mM l-glutamine (Gibco), minimum essential medium nonessential amino acids (Gibco), and 10% FBS (10270, Gibco) at 37 °C, 5% CO_2_. For assays, cells were plated in complete DMEM, 10% FBS at a density of 50,000 cells per well (100 μL per well) in a 96-well plate (Nunc-Immuno MicroWell polystyrene plates with Nunclon delta surface; P8616, Sigma). After 24 h, cells were transfected with Lipofectamine 2000 transfection reagent (Thermo Fisher Scientific) according to the manufacturer’s protocol (0.5 μL per well), with 40 ng pGL3 BRE firefly luciferase plasmid (58), 30 ng pRL-TK *Renilla* luciferase control plasmid (Promega), and 20 ng of empty pHLsec vector control or RGM constructs (or 10 ng of NEO1 plus 10 ng of RGMB constructs) (Fig. 5) in complete DMEM, 0.1% FBS. Twenty-four hours after transfection, media was changed to complete DMEM, 0.1% FBS supplemented with purified BMP2 (3-nM final concentration) or GDF5 (30 nM). After 48-h incubation with BMP2 or GDF5, cells were lysed and the activity of two different luciferases was measured using the Dual-Glo luciferase assay system (Promega). Luminescence was measured using a Luminoskan Ascent luminometer (Labsystems). Fab fragments of anti-BMPR1A antibodies AbD1556 and AbD1564 were produced as described previously (59). Additional BMP2 and GDF5 cellular assays were performed in C3H10T1/2 cells stably transfected with a BMP-responsive luciferase reporter, and ATDC5 cells overexpressing alkaline phosphatase upon stimulation with BMP2/GDF5 (see *SI Appendix* for details).

### MALS.

A SEC-MALS experiment was performed using a Wyatt Dawn HELEOS-II 8-angle light-scattering detector (with 663.8-nm laser) and a Wyatt Optilab rEX refractive index monitor linked to a Shimadzu HPLC system comprising LC-20AD pump, SIL-20A autosampler and SPD20A UV/Vis detector. SEC-MALS of the RGMB_ND_–GDF5–ActR2b_ECD_ complex (1 mg mL^−1^, 100 μL per injection) was performed using Superdex 200 HR 10/30 column equilibrated in 0.5 M NaCl, 20 mM Hepes pH 7.4, 0.5 mL min^−1^ flow rate, 21 °C. GDF5 variant Tyr487Lys/Gln489Asp with increased affinity for the type 2 receptor was used to reconstitute the RGMB_ND_–GDF5–ActR2b_ECD_ complex. SEC-MALS of the RGMB_ND_–GDF5 complex (1 mg mL^−1^, 100 μL per injection) and RGMB_ND_ (1 mg mL^−1^, 100 μL per injection) were performed using a Superdex 200 HR 10/30 column equilibrated either in 0.5 M NaCl, 20 mM Hepes pH 7.5, or 0.5 M NaCl, 20 mM MES pH 5.5 at 0.5 mL min^−1^ flow rate, 21 °C. Scattering data were analyzed and molecular weight was calculated using ASTRA 6 software (Wyatt Technology).

## Supplementary Material

Supplementary File

## References

[1] A. P. Hinck, T. D. Mueller, T. A. Springer, Structural biology and evolution of the TGF-β family. Cold Spring Harb. Perspect. Biol. 8, a022103 (2016). 27638177 10.1101/cshperspect.a022103 PMC5131774

[2] J. L. Wrana, L. Attisano, R. Wieser, F. Ventura, J. Massagué, Mechanism of activation of the TGF-beta receptor. Nature 370, 341–347 (1994). 8047140 10.1038/370341a0

[3] J. T. Thomas., A human chondrodysplasia due to a mutation in a TGF-beta superfamily member. Nat. Genet. 12, 315–317 (1996). 8589725 10.1038/ng0396-315

[4] J. T. Thomas., Disruption of human limb morphogenesis by a dominant negative mutation in CDMP1. Nat. Genet. 17, 58–64 (1997). 9288098 10.1038/ng0997-58

[5] O. Demirhan., A homozygous BMPR1B mutation causes a new subtype of acromesomelic chondrodysplasia with genital anomalies. J. Med. Genet. 42, 314–317 (2005). 15805157 10.1136/jmg.2004.023564 PMC1736042

[6] L. M. Graul-Neumann., Homozygous missense and nonsense mutations in BMPR1B cause acromesomelic chondrodysplasia-type Grebe. Eur. J. Hum. Genet. 22, 726–733 (2014). 24129431 10.1038/ejhg.2013.222 PMC4023204

[7] J. Nickel, A. Kotzsch, W. Sebald, T. D. Mueller, A single residue of GDF-5 defines binding specificity to BMP receptor IB. J. Mol. Biol. 349, 933–947 (2005). 15890363 10.1016/j.jmb.2005.04.015

[8] U. Klammert., GDF-5 can act as a context-dependent BMP-2 antagonist. BMC Biol. 13, 77 (2015). 26385096 10.1186/s12915-015-0183-8 PMC4575486

[9] J. L. Babitt., Repulsive guidance molecule (RGMa), a DRAGON homologue, is a bone morphogenetic protein co-receptor. J. Biol. Chem. 280, 29820–29827 (2005). 15975920 10.1074/jbc.M503511200

[10] T. A. Samad., DRAGON, a bone morphogenetic protein co-receptor. J. Biol. Chem. 280, 14122–14129 (2005). 15671031 10.1074/jbc.M410034200

[11] J. L. Babitt., Bone morphogenetic protein signaling by hemojuvelin regulates hepcidin expression. Nat. Genet. 38, 531–539 (2006). 16604073 10.1038/ng1777

[12] E. Corradini, J. L. Babitt, H. Y. Lin, The RGM/DRAGON family of BMP co-receptors. Cytokine Growth Factor Rev. 20, 389–398 (2009). 19897400 10.1016/j.cytogfr.2009.10.008 PMC3715994

[13] C. Siebold, T. Yamashita, P. P. Monnier, B. K. Mueller, R. J. Pasterkamp, RGMs: Structural insights, molecular regulation, and downstream signaling. Trends Cell Biol. 27, 365–378 (2017). 28007423 10.1016/j.tcb.2016.11.009 PMC5404723

[14] V. S. Li., Frequent inactivation of axon guidance molecule RGMA in human colon cancer through genetic and epigenetic mechanisms. Gastroenterology 137, 176–187 (2009). 19303019 10.1053/j.gastro.2009.03.005

[15] E. Demicheva., Targeting repulsive guidance molecule A to promote regeneration and neuroprotection in multiple sclerosis. Cell Rep. 10, 1887–1898 (2015). 25801027 10.1016/j.celrep.2015.02.048

[16] C. H. Bell., Structure of the repulsive guidance molecule (RGM)-neogenin signaling hub. Science 341, 77–80 (2013). 23744777 10.1126/science.1232322 PMC4730555

[17] E. G. Healey., Repulsive guidance molecule is a structural bridge between neogenin and bone morphogenetic protein. Nat. Struct. Mol. Biol. 22, 458–465 (2015). 25938661 10.1038/nsmb.3016 PMC4456160

[18] D. H. Lee., Neogenin inhibits HJV secretion and regulates BMP-induced hepcidin expression and iron homeostasis. Blood 115, 3136–3145 (2010). Correction in: *Blood* **116**, 151 (2010). 20065295 10.1182/blood-2009-11-251199 PMC2858467

[19] F. W. Huang, J. L. Pinkus, G. S. Pinkus, M. D. Fleming, N. C. Andrews, A mouse model of juvenile hemochromatosis. J. Clin. Invest. 115, 2187–2191 (2005). 16075059 10.1172/JCI25049 PMC1180543

[20] V. Niederkofler, R. Salie, S. Arber, Hemojuvelin is essential for dietary iron sensing, and its mutation leads to severe iron overload. J. Clin. Invest. 115, 2180–2186 (2005). 16075058 10.1172/JCI25683 PMC1180556

[21] G. Papanikolaou., Mutations in HFE2 cause iron overload in chromosome 1q-linked juvenile hemochromatosis. Nat. Genet. 36, 77–82 (2004). 14647275 10.1038/ng1274

[22] S. Canali, C. Y. Wang, K. B. Zumbrennen-Bullough, A. Bayer, J. L. Babitt, Bone morphogenetic protein 2 controls iron homeostasis in mice independent of Bmp6. Am. J. Hematol. 92, 1204–1213 (2017). 28815688 10.1002/ajh.24888 PMC5986189

[23] Z. Zhou., Neogenin regulation of BMP-induced canonical Smad signaling and endochondral bone formation. Dev. Cell 19, 90–102 (2010). 20643353 10.1016/j.devcel.2010.06.016 PMC2924163

[24] E. E. Storm., Limb alterations in brachypodism mice due to mutations in a new member of the TGF beta-superfamily. Nature 368, 639–643 (1994). 8145850 10.1038/368639a0

[25] C. Tian., The neogenin/DCC homolog UNC-40 promotes BMP signaling via the RGM protein DRAG-1 in C. elegans. Development 140, 4070–4080 (2013). 24004951 10.1242/dev.099838 PMC3775419

[26] A. Kotzsch, J. Nickel, A. Seher, W. Sebald, T. D. Müller, Crystal structure analysis reveals a spring-loaded latch as molecular mechanism for GDF-5-type I receptor specificity. EMBO J. 28, 937–947 (2009). 19229295 10.1038/emboj.2009.37 PMC2670865

[27] C. Lanzara., Spectrum of hemojuvelin gene mutations in 1q-linked juvenile hemochromatosis. Blood 103, 4317–4321 (2004). 14982873 10.1182/blood-2004-01-0192

[28] P. H. Francis-West, J. Parish, K. Lee, C. W. Archer, BMP/GDF-signalling interactions during synovial joint development. Cell Tissue Res. 296, 111–119 (1999). 10199971 10.1007/s004410051272

[29] R. Merino., Expression and function of Gdf-5 during digit skeletogenesis in the embryonic chick leg bud. Dev. Biol. 206, 33–45 (1999). 9918693 10.1006/dbio.1998.9129

[30] R. Chijimatsu, T. Saito, Mechanisms of synovial joint and articular cartilage development. Cell. Mol. Life Sci. 76, 3939–3952 (2019). 31201464 10.1007/s00018-019-03191-5 PMC11105481

[31] K. M. Lyons, V. Rosen, BMPs, TGFβ, and border security at the interzone. Curr. Top. Dev. Biol. 133, 153–170 (2019). 30902251 10.1016/bs.ctdb.2019.02.001

[32] Q. Wu, C. C. Sun, H. Y. Lin, J. L. Babitt, Repulsive guidance molecule (RGM) family proteins exhibit differential binding kinetics for bone morphogenetic proteins (BMPs). PLoS One 7, e46307 (2012). 23029472 10.1371/journal.pone.0046307 PMC3459908

[33] K. Heinecke., Receptor oligomerization and beyond: A case study in bone morphogenetic proteins. BMC Biol. 7, 59 (2009). 19735544 10.1186/1741-7007-7-59 PMC2749821

[34] Y. Xia., Repulsive guidance molecule RGMa alters utilization of bone morphogenetic protein (BMP) type II receptors by BMP2 and BMP4. J. Biol. Chem. 282, 18129–18140 (2007). 17472960 10.1074/jbc.M701679200

[35] M. Hagihara., Neogenin, a receptor for bone morphogenetic proteins. J. Biol. Chem. 286, 5157–5165 (2011). 21149453 10.1074/jbc.M110.180919 PMC3037628

[36] C. S. Hill, Spatial and temporal control of NODAL signaling. Curr. Opin. Cell Biol. 51, 50–57 (2018). 29153705 10.1016/j.ceb.2017.10.005

[37] H. Tian, K. Mythreye, C. Golzio, N. Katsanis, G. C. Blobe, Endoglin mediates fibronectin/α5β1 integrin and TGF-β pathway crosstalk in endothelial cells. EMBO J. 31, 3885–3900 (2012). 22940691 10.1038/emboj.2012.246 PMC3463850

[38] T. Saito., Structural basis of the human endoglin-BMP9 interaction: Insights into BMP signaling and HHT1. Cell Rep. 19, 1917–1928 (2017). 28564608 10.1016/j.celrep.2017.05.011 PMC5464963

[39] N. G. Tassew., Modifying lipid rafts promotes regeneration and functional recovery. Cell Rep. 8, 1146–1159 (2014). 25127134 10.1016/j.celrep.2014.06.014

[40] L. Silvestri, A. Pagani, C. Camaschella, Furin-mediated release of soluble hemojuvelin: A new link between hypoxia and iron homeostasis. Blood 111, 924–931 (2008). 17938254 10.1182/blood-2007-07-100677

[41] A. J. Mothe., RGMa inhibition with human monoclonal antibodies promotes regeneration, plasticity and repair, and attenuates neuropathic pain after spinal cord injury. Sci. Rep. 7, 10529 (2017). 28874746 10.1038/s41598-017-10987-7 PMC5585220

[42] P. Böser., Anti-repulsive guidance molecule C (RGMc) antibodies increases serum iron in rats and cynomolgus monkeys by hepcidin downregulation. AAPS J. 17, 930–938 (2015). 25896304 10.1208/s12248-015-9770-4 PMC4476998

[43] S. Kovac., Anti-hemojuvelin antibody corrects anemia caused by inappropriately high hepcidin levels. Haematologica 101, e173–e176 (2016). 26944476 10.3324/haematol.2015.140772 PMC5004359

[44] S. V. Torti, E. Lemler, B. K. Mueller, A. Popp, F. M. Torti, Effects of anti-repulsive guidance molecule C (RGMc/Hemojuvelin) antibody on hepcidin and iron in mouse liver and tumor xenografts. Clin. Exp. Pharmacol. 6, 223 (2016). 28203489 10.4172/2161-1459.1000223 PMC5305030

[45] S. R. Goulding, A. M. Sullivan, G. W. O’Keeffe, L. M. Collins, The potential of bone morphogenetic protein 2 as a neurotrophic factor for Parkinson’s disease. Neural Regen. Res. 15, 1432–1436 (2020). 31997802 10.4103/1673-5374.274327 PMC7059567

[46] F. M. Hurley, D. J. Costello, A. M. Sullivan, Neuroprotective effects of delayed administration of growth/differentiation factor-5 in the partial lesion model of Parkinson’s disease. Exp. Neurol. 185, 281–289 (2004). 14736509 10.1016/j.expneurol.2003.10.003

[47] S. V. Hegarty, G. W. O’Keeffe, A. M. Sullivan, Neurotrophic factors: From neurodevelopmental regulators to novel therapies for Parkinson’s disease. Neural Regen. Res. 9, 1708–1711 (2014). 25422631 10.4103/1673-5374.143410 PMC4238158

[48] A. R. Aricescu, W. Lu, E. Y. Jones, A time- and cost-efficient system for high-level protein production in mammalian cells. Acta Crystallogr. D Biol. Crystallogr. 62, 1243–1250 (2006). 17001101 10.1107/S0907444906029799

[49] T. S. Walter., A procedure for setting up high-throughput nanolitre crystallization experiments. Crystallization workflow for initial screening, automated storage, imaging and optimization. Acta Crystallogr. D Biol. Crystallogr. 61, 651–657 (2005). 15930615 10.1107/S0907444905007808 PMC7159505

[50] T. S. Walter., Lysine methylation as a routine rescue strategy for protein crystallization. Structure 14, 1617–1622 (2006). 17098187 10.1016/j.str.2006.09.005 PMC7126202

[51] W. Kabsch, Integration, scaling, space-group assignment and post-refinement. Acta Crystallogr. D Biol. Crystallogr. 66, 133–144 (2010). 20124693 10.1107/S0907444909047374 PMC2815666

[52] P. R. Evans, G. N. Murshudov, How good are my data and what is the resolution? Acta Crystallogr. D Biol. Crystallogr. 69, 1204–1214 (2013). 23793146 10.1107/S0907444913000061 PMC3689523

[53] G. Winter, C. M. Lobley, S. M. Prince, Decision making in xia2. Acta Crystallogr. D Biol. Crystallogr. 69, 1260–1273 (2013). 23793152 10.1107/S0907444913015308 PMC3689529

[54] A. J. McCoy., Phaser crystallographic software. J. Appl. Cryst. 40, 658–674 (2007). 19461840 10.1107/S0021889807021206 PMC2483472

[55] P. Emsley, B. Lohkamp, W. G. Scott, K. Cowtan, Features and development of Coot. Acta Crystallogr. D Biol. Crystallogr. 66, 486–501 (2010). 20383002 10.1107/S0907444910007493 PMC2852313

[56] P. V. Afonine., Towards automated crystallographic structure refinement with phenix.refine. Acta Crystallogr. D Biol. Crystallogr. 68, 352–367 (2012). 22505256 10.1107/S0907444912001308 PMC3322595

[57] C. J. Williams., MolProbity: More and better reference data for improved all-atom structure validation. Protein Sci. 27, 293–315 (2018). 29067766 10.1002/pro.3330 PMC5734394

[58] O. Korchynskyi, P. ten Dijke, Identification and functional characterization of distinct critically important bone morphogenetic protein-specific response elements in the Id1 promoter. J. Biol. Chem. 277, 4883–4891 (2002). 11729207 10.1074/jbc.M111023200

[59] S. Harth, A. Kotzsch, J. Hu, W. Sebald, T. D. Mueller, A selection fit mechanism in BMP receptor IA as a possible source for BMP ligand-receptor promiscuity. PLoS One 5, e13049 (2010). 20927405 10.1371/journal.pone.0013049 PMC2946932

