# Supplementary material for: Repulsive guidance molecules lock growth differentiation factor 5 in an inhibitory complex
Source: Proc Natl Acad Sci U S A. 2020 Jun 23;117(27):15620–31. doi: 10.1073/pnas.2000561117 (PMC7354924; doi:10.1073/pnas.2000561117)
Supplement: Supplementary File [file pnas.2000561117.sapp.pdf]

PNAS
www.pnas.org

Supplementary Information for

Repulsive guidance molecules lock growth differentiation factor 5 in an inhibitory complex

Tomas Malinauskas[1,*], Tina V. Peer[2], Benjamin Bishop[1], Thomas D. Mueller[2,*], Christian Siebold[1,*]

[1]Division of Structural Biology, Wellcome Centre for Human Genetics, University of Oxford, Roosevelt Drive, OX3 7BN, Oxford, United Kingdom.

[2]Department of Molecular Plant Physiology and Biophysics, Julius-von-Sachs Institute, University of Würzburg, Julius-von-Sachs-Platz 2, 97082, Würzburg, Germany.

[*]Correspondence should be addressed to T.M. (tomas@strubi.ox.ac.uk), T.D.M. (mueller@biozentrum.uni-wuerzburg.de) or C.S. (christian@strubi.ox.ac.uk).

This PDF file includes:

Supplementary Materials and Methods
Table S1
Figures S1 to S12
Supplementary References

www.pnas.org/cgi/doi/10.1073/pnas.2000561117

Supplementary Materials and Methods

Production of RGMB in insect cells for binding studies in the presence of lower concentration (0.005%) of Tween 20 (Fig. S8).

Soluble mature RGMB (lacking the unpaired cysteine at the N-terminus Cys48 and the C-terminal Asp413, which is modified to carry the GPI anchor for membrane attachment) was produced in a baculovirus-transfected insect cell expression system. The gene encoding residues Gln49 to Gly412 of human RGMB was cloned into a modified pBAC3 expression vector (Novagen). The expression construct comprises an N-terminal gp64 signal peptide followed by a hexahistidine sequence and a thrombin recognition/cleavage site. Recombinant baculovirus was generated by homologous recombination in Sf9 insect cells; 250 ng of pBAC3-RGMB expression vector was mixed with 50 ng triple cut linearized BACVECTOR 3000 virus DNA (Novagen) and the mixture was transfected into Sf9 cells adapted to serum-free growth medium (Insect Xpress, Lonza) using the transfection reagent Insect Gene Juice (Novagen) following the manufacturer's recommendation. Cells were grown at 27 °C for five days. Six clones of recombinant baculovirus were selected by performing a plaque assay, the virus of these clones were amplified for two rounds in adherent growing Sf9 cells and the clone exhibiting the highest expression for RGMB was selected by Western blot analysis using an anti-His-tag antibody (Penta-HIS-HRP, 5Prime). Recombinant virus of this clone was then amplified in two further rounds using suspension-growing Sf9 insect cells (TriEx, Novagen) growing in serum-free medium (Insect Xpress, Lonza) at 27°C and 90 rpm. Virus was harvested by centrifugation (5 min, 400g, 21 °C) and stored at 4 °C in the dark until further use. For RGMB expression, BTI-Tn-5B1-4 insect cells (HighFive, Novagen), which were grown in suspension in serum-free medium (IPL41 (HyClone) supplemented with 1% (v/v) 100× Lipid-Mix (Sigma), 100 U/ml penicillin-G and 100 µg/ml streptomycin), were seeded at a density of 1.5×10^6 cells/ml and infected with the recombinant baculovirus (from a fourth or higher virus amplification round) using a multiplicity of infection (MOI) of 5. After incubating the cells for 4 to 5 days at 27 °C and 90 rpm, the supernatant was harvested, clarified by centrifugation and dialyzed three times against 10 volumes 50 mM sodium phosphate pH 8.3, 300 mM NaCl, 10 mM imidazole (buffer A). RGMB protein was purified from the supernatant by immobilized metal ion affinity chromatography (IMAC) using His Buster Nickel Affinity Gel (Amocol). After washing the affinity resin with 20 column volumes of buffer A, RGMB protein was eluted with buffer A supplemented with 500 mM imidazole. RGMB containing fractions were pooled and dialyzed against 100 volumes 20 mM Tris-HCl pH 8.0, 35 mM NaCl and subjected to anion exchange chromatography employing a HiTrap QXL column (GE Healthcare) and a linear gradient from 0.04 to 0.2 M NaCl in 20 mM Tris-HCl pH 8.3. Fractions containing pure RGMB protein were pooled, dialyzed against 1 mM HCl and freeze-dried.

Production of full-length extracellular domains (ECDs) of RGMA, RGMB and RGMC in FreeStyle293 cells for binding studies (Fig. S9).

Soluble full-length extracellular domains (ECDs) of RGMs for SPR analysis shown in Fig. S9 were obtained from transient expression in FreeStyle 293 cells. The soluble domain of RGMA, B and C comprising residues Cys48 to Arg423 (RGMA), residues Gln49 to Gly412 (RGMB), and residues Gln36 to Asp400 (RGMC) were cloned between the AgeI and KpnI endonuclease restriction sites of the expression vector pHLsec (1). All C-terminal residues past the above-mentioned residues were removed to impede membrane-anchoring due to lipidification. Plasmid DNA was amplified in NovaBlue cells transformed with the respective vector DNA and isolated using a QIAGEN Plasmid Plus Midi kit according to the manufacturer's recommendation. HEK293-F (FreeStyle293) cells were grown at 37 °C, 8% CO_2, 60% humidity and 100 rpm in FreeStyle 293 expression medium (Gibco) supplemented with 1% (v/v) PenStrep (Lonza). Plasmid DNA was transiently transfected into HEK293-F cells using polyethyleneimine (PEI) (2). For transfection FreeStyle293 cells were diluted to a cell density of 0.75×10^6 cells/ml. Two solutions were prepared: for each ml of cell culture, 35 µl Opti-MEM I medium (Gibco) containing 1 µg vector DNA (solution A) and 35 µl Opti-MEM medium containing 2 µg PEI (25 kDa linear, Polyscience) were prepared. Both solutions were mixed, incubated at 21°C for 25 min and slowly added to the cell culture. Typical volumes for transient expression of RGM proteins in FreeStyle 293 cells were approximately 800 ml. After five days, cell culture medium was clarified by centrifugation (800×g, 4 °C, 25 min) and the supernatant was dialyzed twice against 20 volumes 50 mM sodium phosphate, 300 mM NaCl pH 8.3 (running buffer). The dialyzed cell culture supernatant was submitted to immobilized metal ion affinity chromatography employing a 5ml HisTrap excel column (GE Healthcare). The column was washed with running buffer until UV absorbance at 280 nm reached background levels. Then RGM proteins were eluted in a step gradient with running buffer supplemented with 500 mM imidazole. Protein-containing fractions were identified by SDS-PAGE, pooled and dialyzed against 1 mM HCl. As second purification step to remove residual protein impurities a reversed-phase HPLC chromatography was performed. Dialyzed RGM protein solutions were submitted to a C4 reserved-phase HPLC column (4.6 × 250 mm Jupiter C4, Phenomenex) and eluted with a non-linear gradient from 0.1% (v/v) trifluoroacetic acid (TFA) in water to 100% acetonitrile. Fractions containing pure RGM proteins were identified by SDS-PAGE, pooled and freeze-dried.

Cellular assays of BMP2 and GDF5 signaling in C3H10T1/2 cells.

The murine mesenchymal stem cell line C3H10T1/2 stably transfected with a BRE-Luc reporter expression construct (gift from P. ten Dijke, Leiden; (3)) was used to determine the half-maximal effective concentration of BMP2 and GDF5 for SMAD1/5/8 activation as well as its RGM-mediated inhibition. C3H10T1/2-BRE cells were grown at 37 °C, 5% CO_2 in DMEM medium (Gibco) supplemented with 10% (v/v) fetal calf serum (Biochrom), 100 U/ml penicillin G, 100 µg/ml streptomycin and 0.2 mg/ml G418 sulfate (Sigma). In a 96 well micro titer plate 7×10^3 C3H10T1/2-BRE cells were seeded per well and the cells were incubated at 37 °C, 5% CO_2 for 16 h. Cell medium was replaced by starvation medium (the above-described DMEM medium but

supplemented with 0.1% (v/v) fetal calf serum). To determine the half-maximal effective concentrations of BMP2 and GDF5 a log2 or log3 dilution series of the growth factor (typical range was 0.01 nM to 300 nM) was added to the cell suspension and the cells were incubated for 72 h at 37 °C and 5 % CO_2. Thereafter, medium was removed and cells were washed with PBS. Cell lysis was performed by addition of 50 µl reporter lysis buffer (Promega) and freezing the cells for 30 min at -80 °C. After thawing, 30 µl of each lysate was transferred into a new microtiter plate. Each well was mixed with 50 µl substrate of the luciferase assay system (Promega) and light emission was quantified using a luminometer system Luminoskan Ascent (Thermo Fisher). Each experiment was performed in duplicate on a single micro titer plate. To determine the effect of soluble RGMB on BMP2- or GDF5-induced SMAD signaling, C3H10T1/2-BRE cells were stimulated with 10 nM BMP2 or GDF5 in the presence of a log3 or log2 dilution series of soluble RGMB starting with a highest concentration of 2000 nM (in the case of BMP2) and 1500 nM (in the case of GDF5). Stimulation with 10 nM BMP2 and GDF5 without addition of soluble RGMB was used as a control, as well as a log3 or log2 dilution of soluble RGMB without stimulation by BMP2 or GDF5. Luciferase expression was analyzed as describe above to determine the half-maximal effective concentration of both growth factors. Data were analyzed using KaleidaGraph 4.5 (GraphPad) employing a non-linear regression analysis (IC50 fitting routine supplied by GraphPad). Using EC_{50} and IC_{50} values obtained from both assays, the inhibitory constant for RGMB was calculated using the Cheng-Prusoff equation (4).

Cellular assays of BMP2 and GDF5 signaling in ATDC5 cells.
ATDC5 cells (RIKEN No. RCB0565) were grown at 37 °C, 5% CO_2 in DMEM/F12 medium (Gibco) supplemented with 100 U/ml penicillin G, 100 µg/ml streptomycin and 5% (v/v) heat-inactivated fetal calf serum (FCS, Biochrome). Prior to the assay, cells were seeded in 96 well plates at a density of 1.2×10^5 cells/ml and grown for 16 h at 37 °C, 5% CO_2. Thereafter the medium was removed and replaced with DMEM/F12 starvation medium supplemented with 2% FCS. To determine half-maximal effective concentrations (EC_{50}) of BMP/GDF proteins a log2 or log3 dilution series (typical concentration range 0.01 nM to 300 nM) of the respective growth factor was added. For assays to determine the half-maximal inhibitory concentrations (IC_{50}) of a competitor, a log3 dilution series of the competitor (e.g. RGMB) supplemented with a constant concentration of the growth factor was added. Cells were incubated at 37 °C, 5% CO_2 for 72 h. Thereafter medium was removed, cells were washed with sterile phosphate-buffered saline and subsequently lysed for 30 min by adding 0.1 M glycine pH 9.6, 1% (v/v) NP40, 1 mM $MgCl_2$, 1 mM $ZnCl_2$. For measurement of alkaline phosphatase activity para-nitrophenylphosphate (PNPP) solution (Thermo Fisher) was added according to the manufacturer's recommendation. After incubation for 5 to 10 min at room temperature absorbance at 405 nm and 630 nm (background) was measured using a plate reader. All measurements were performed in duplicate on a single plate. For assays to determine the EC_{50} of the respective growth factor wells containing medium without growth factor added served as a negative control. For experiments to determine the half-maximal inhibitory concentrations (IC_{50}) of a competitor, e.g. RGMB, a positive control (cells

stimulated with the same concentration of the growth factor but without competitor) and a negative control (cells without growth factor or competitor added) were measured on the same microtiter plate. Transfection efficiency of ATDC5 and C3H10T1/2 cell lines was too low to perform studies of full length RGMs in these cells. Data were analyzed using KaleidaGraph 4.5 (GraphPad) employing a non-linear regression analysis (IC50 fitting routine supplied by GraphPad).

RGM and NEO1 constructs for cellular assays and Western blot analysis.
For cellular assays and Western blot analysis, RGM constructs were cloned into the pHLsec vector (1) with an N-terminal His_6-tag (for detection) and no C-terminal tags (to avoid any interference with the GPI-mediated anchoring on the cell surface). All constructs started with a secretion signal from the pHLsec vector, followed by a Glu-Thr-Gly-His_6-Gly-Ser-Gly sequence and the target protein (full-length RGMB: Gln50–Leu437; $RGMB_{\Delta N}$: Asp137–Leu437; full-length RGMA: Ser46–Cys450; $RGMA_{\Delta N}$: Asp140–Cys450; full-length RGMC: Gln36–Gln426; $RGMC_{\Delta N}$: Asp146–Gln426). The full-length NEO1 construct contained a secretion signal derived from the pHLsec vector, followed by Glu-Thr-Gly and mouse NEO1 (isoform 2 precursor, NP_001036217.1) residues Ala42–Ala1465, a single Gly, and a Rhodopsin 1D4 tag (TETSQVAPA) (5). The $NEO1_{\Delta C}$ construct contained a N-terminal secretion signal derived from the pHLsec vector, followed by Glu-Thr-Gly-Ser-Gly-Ser-Ala, mouse NEO1 (isoform 2 precursor, NP_001036217.1) residues Pro51–Asn1107, a Gly-Ser-Gly-Gly linker, a monomerized transmembrane helix of human Glycophorin-A (Ala84–Lys120 with mutations Gly98Leu, Gly102Leu, P02724; (6)), a 3C protease cleavage site, mono Venus (Met1–Lys239, GenBank ID AIN46602.1), and a C-terminal His_8-tag.

HEK 293T cells (ATCC CRL-11268) were grown in DMEM high glucose media (D6546, Sigma) supplemented with 2 mM L-glutamine (Gibco), minimum essential medium non-essential amino acids (Gibco) and 10% FBS (10270, Gibco) at 37 °C, 5% CO_2. For assays, cells were plated in complete DMEM, 10% FBS at a density of ~2 million cells/well (2 ml/well) in a 6-well plate (Greiner Bio-One, 657160). After 24 h, cells were transfected using Lipofectamine 2000 transfection reagent (Thermo Fisher Scientific) according to the manufacturer's protocol (12.5 µl Lipofectamine plus 2.5 µg pHLsec-RGM DNA per well) and grown in complete DMEM, 2% FBS for 72 h. Conditioned media and cell lysates (lysis buffer 150 mM NaCl, 20 mM Tris-HCl pH 8.0, 5 mM EDTA pH 8.0, 10 mM $MgCl_2$, 1 mM PMSF, 1% Triton X-100) were reduced with β-mercaptoethanol (286 mM), heated (95 °C, 10 min) and resolved using SDS-PAGE (NuPAGE 4–12% Bis-Tris protein gels, Thermo Fisher Scientific), followed by a transfer to nitrocellulose membranes (0.45 µm, Amersham), blocking in PBS, 5% skimmed milk and probing with antibodies. Primary antibodies: mouse monoclonal anti-His_5 (Qiagen, 34660), rabbit polyclonal anti-Cyclophilin A (Abcam, ab41684). Secondary antibodies: goat polyclonal anti-mouse IgG (Fc specific) conjugated to horseradish peroxidase (Sigma, A0168), goat polyclonal anti-rabbit IgG H&L conjugated to peroxidase (Abcam, ab97051). Peroxidase was visualized by

chemiluminescence using the Clarity Western ECL Substrate (Bio-Rad, 1705060) and ChemiDoc MP imaging system (Bio-Rad).

Table S1. Summary of binding constants.

**Binding constants derived in presence of a low concentration of detergent (0.005% (v/v) Tween 20)***				
Ligand ($RU_{immobilized}$)	Analyte	k_{on}, M^{-1} s^{-1}	k_{off}, s^{-1}	K_d, nM
BMP2 (570)	$RGMB_{ECD}$	$(5.3 \pm 2) \times 10^6$	$(11 \pm 3) \times 10^{-3}$	4.2 ± 1.2
GDF5 (470)	$RGMB_{ECD}$	$(1.8 \pm 0.2) \times 10^6$	$(2.5 \pm 0.5) \times 10^{-3}$	2.7 ± 0.9
BMP2 (570)	BMPR1A	$(1.1 \pm 0.1) \times 10^5$	$(2.7 \pm 0.1) \times 10^{-3}$	59 ± 29
GDF5 (470)	BMPR1A	$(0.9 \pm 0.02) \times 10^5$	$(4.8 \pm 0.03) \times 10^{-3}$	125 ± 3
GDF5 (500)	$RGMA_{ECD}$**	$(4.2 \pm 0.9) \times 10^5$	$(5.2 \pm 1.3) \times 10^{-4}$	1.3 ± 0.5
GDF5 (500)	$RGMB_{ECD}$**	$(4.2 \pm 0.2) \times 10^5$	$(1.6 \pm 0.1) \times 10^{-3}$	3.7 ± 0.4
GDF5 (500)	$RGMC_{ECD}$**	$(2.4 \pm 0.5) \times 10^5$	$(6.4 \pm 1.5) \times 10^{-4}$	2.8 ± 1.2
GDF5 Arg438Ala (702)	$RGMA_{ECD}$	$(2.7 \pm 0.7) \times 10^5$	$(4.5 \pm 0.5) \times 10^{-4}$	1.7 ± 0.6
GDF5 Arg438Ala (702)	$RGMB_{ECD}$	$(1.0 \pm 0.2) \times 10^6$	$(1.5 \pm 0.2) \times 10^{-3}$	1.5 ± 0.3
GDF5 Arg438Ala (702)	$RGMC_{ECD}$	$(1.6 \pm 0.3) \times 10^5$	$(2.6 \pm 0.02) \times 10^{-4}$	2.3 ± 0.5
GDF5 Arg438Leu (977)	$RGMA_{ECD}$	$(3.0 \pm 0.7) \times 10^5$	$(4.2 \pm 0.5) \times 10^{-4}$	1.4 ± 0.5
GDF5 Arg438Leu (977)	$RGMB_{ECD}$	$(1.0 \pm 0.2) \times 10^6$	$(1.1 \pm 0.1) \times 10^{-3}$	1.1 ± 0.2
GDF5 Arg438Leu (977)	$RGMC_{ECD}$	$(1.9 \pm 0.2) \times 10^5$	$(4.1 \pm 0.3) \times 10^{-4}$	2.2 ± 0.5
BMP2 (570)	ActR2b	n.d.	n.d.	2700 ± 300
GDF5 (470)	ActR2b	n.d.	n.d.	1300 ± 200
TGF-β2 (300)	$RGMB_{ECD}$	n.d.	n.d.	No binding
Activin A (350)	$RGMB_{ECD}$	n.d.	n.d.	No binding
**Binding constants derived in presence of a high concentration of detergent (0.05% (v/v) Tween 20)*****				
GDF5 (1370)	$RGMB_{ECD}$	n.d.	n.d.	8800
GDF5 (970)	$RGMB_{ND}$	n.d.	n.d.	2700
GDF5 (970)	$RGMA_{ND}$	n.d.	n.d.	16700
GDF5 (970)	$RGMC_{ND}$	n.d.	n.d.	3900
GDF5 (1370)	$RGMC_{ND}$ Gly99Arg	n.d.	n.d.	> 20000
GDF5 (1370)	$RGMB_{ND}$ Gly101Arg	n.d.	n.d.	> 150000
GDF5 (970)	$RGMB_{ND}$ Leu103Glu	n.d.	n.d.	No binding
GDF5 (1370)	$RGMB_{ND}$ His106Arg	n.d.	n.d.	> 200000
BMP2 (960)	$RGMB_{ND}$	n.d.	n.d.	20100
GDF5 (830)	$RGMB_{ND}$	n.d.	n.d.	1800
GDF5 (830)	$RGMB_{ND}$ His106Leu	n.d.	n.d.	> 200000
GDF5 (830)	$RGMB_{ND}$ His106Asn	n.d.	n.d.	> 150000
GDF5 (1370)	Bovine serum albumin	n.d.	n.d.	No binding

Table S1. Summary of binding constants (legend).

n.d. not determined.

* binding constants for the interaction of BMP ligands with RGM and type-1 receptor were derived by fitting the association and dissociation phase of the SPR sensorgram employing a Langmuir type 1:1 interaction to yield kinetic rate constants k_{on} and k_{off}. Equilibrium binding constants K_d was then calculated from the equation $K_d = k_{off}/k_{on}$. Since the interaction of BMP ligands with type-2 receptors exhibits a fast binding kinetics, which cannot be analyzed from the SPR sensorgrams, binding affinities (K_d) were derived by evaluating the dose-dependency of the equilibrium binding.

** RGM proteins were recombinantly derived from transient expression in FreeStyle293 cells grown in suspension-adapted cell culture.

*** binding affinities (K_d) were derived from the dose-dependency of equilibrium binding.

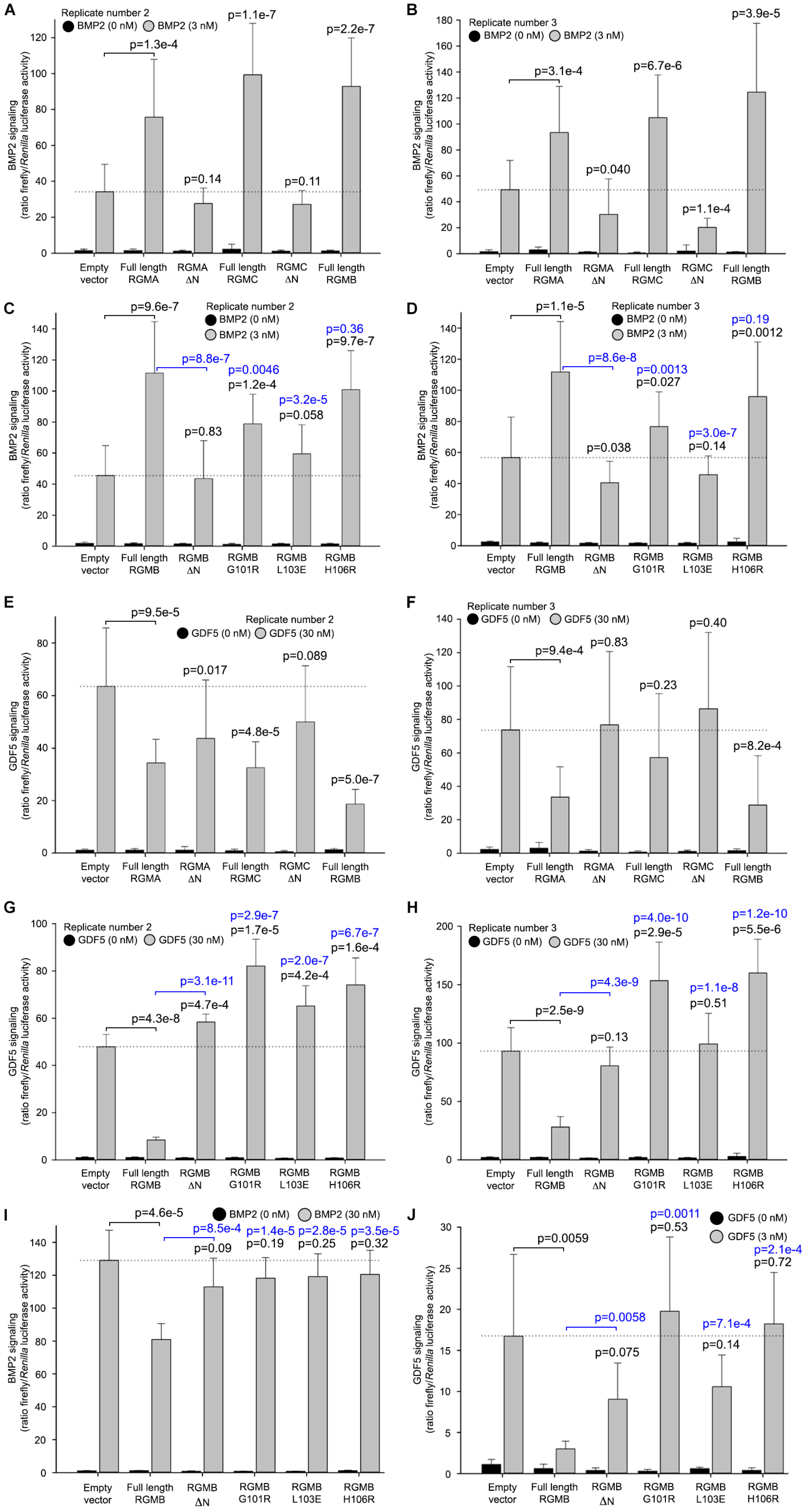
A
Replicate number 2
BMP2 (0 nM) BMP2 (3 nM)
BMP2 signaling
(ratio firefly/Renilla luciferase activity)
p=1.3e-4
p=1.1e-7
p=2.2e-7
p=0.14
p=0.11
Empty vector
Full length RGMA
RGMA ΔN
Full length RGMC
RGMC ΔN
Full length RGMB
B
Replicate number 3
BMP2 (0 nM) BMP2 (3 nM)
p=3.1e-4
p=6.7e-6
p=3.9e-5
p=0.040
p=1.1e-4
Empty vector
Full length RGMA
RGMA ΔN
Full length RGMC
RGMC ΔN
Full length RGMB
C
Replicate number 2
BMP2 (0 nM)
BMP2 (3 nM)
p=9.6e-7
p=8.8e-7
p=0.0046
p=1.2e-4
p=3.2e-5
p=0.058
p=0.36
p=9.7e-7
p=0.83
Empty vector
Full length RGMB
RGMB ΔN
RGMB G101R
RGMB L103E
RGMB H106R
D
Replicate number 3
BMP2 (0 nM)
BMP2 (3 nM)
p=1.1e-5
p=8.6e-8
p=0.0013
p=0.027
p=0.19
p=0.0012
p=0.038
p=3.0e-7
p=0.14
Empty vector
Full length RGMB
RGMB ΔN
RGMB G101R
RGMB L103E
RGMB H106R
E
Replicate number 2
GDF5 (0 nM) GDF5 (30 nM)
GDF5 signaling
(ratio firefly/Renilla luciferase activity)
p=9.5e-5
p=0.017
p=0.089
p=4.8e-5
p=5.0e-7
Empty vector
Full length RGMA
RGMA ΔN
Full length RGMC
RGMC ΔN
Full length RGMB
F
Replicate number 3
GDF5 (0 nM) GDF5 (30 nM)
p=9.4e-4
p=0.83
p=0.40
p=0.23
p=8.2e-4
Empty vector
Full length RGMA
RGMA ΔN
Full length RGMC
RGMC ΔN
Full length RGMB
G
Replicate number 2
GDF5 (0 nM) GDF5 (30 nM)
p=2.9e-7
p=1.7e-5
p=6.7e-7
p=1.6e-4
p=2.0e-7
p=4.2e-4
p=3.1e-11
p=4.7e-4
p=4.3e-8
Empty vector
Full length RGMB
RGMB ΔN
RGMB G101R
RGMB L103E
RGMB H106R
H
Replicate number 3
GDF5 (0 nM) GDF5 (30 nM)
p=4.0e-10
p=2.9e-5
p=1.2e-10
p=5.5e-6
p=4.3e-9
p=1.1e-8
p=0.51
p=2.5e-9
p=0.13
Empty vector
Full length RGMB
RGMB ΔN
RGMB G101R
RGMB L103E
RGMB H106R
I
BMP2 (0 nM) BMP2 (30 nM)
p=4.6e-5
p=8.5e-4
p=0.09
p=1.4e-5
p=0.19
p=2.8e-5
p=0.25
p=3.5e-5
p=0.32
Empty vector
Full length RGMB
RGMB ΔN
RGMB G101R
RGMB L103E
RGMB H106R
J
GDF5 (0 nM)
GDF5 (3 nM)
p=0.0059
p=0.0011
p=0.53
p=2.1e-4
p=0.72
p=0.0058
p=0.075
p=7.1e-4
p=0.14
Empty vector
Full length RGMB
RGMB ΔN
RGMB G101R
RGMB L103E
RGMB H106R

Fig. S1. Replicates of BMP2- and GDF5-responsive cellular assay of RGM function in kidney cells (LLC-PK1).

A and **B**, two replicates of RGM–BMP2 signaling assay presented in **Fig 1B**. **C** and **D**, two replicates of RGMB–BMP2 signaling assay presented in **Fig. 1D**. **E** and **F**, two replicates of RGM–GDF5 signaling assay presented in **Fig. 1C**. **G** and **H**, two replicates of RGMB–GDF5 signaling assay presented in **Fig. 1E**. **I**, RGMB–BMP2 signaling assay in the presence of higher concentration of BMP2 (30 nM) than presented in **Fig. 1B** and **D,** and **Fig. S1A**–**D** (3 nM BMP2) showing that at a higher concentration of BMP2, the effect of RGMB-mediated potentiation of BMP2 signaling is not detectable in this assay. **J**, RGMB–GDF5 signaling assay in the presence of a lower concentration of GDF5 (3 nM) than presented in **Fig. 1C** and **E** and **Fig. S1E**–**H** (30 nM GDF5) showing that at a lower GDF5 concentration, signaling levels are lower (as well as the effect of RGMB-mediated inhibition of GDF5) and varies more as indicated by higher standard deviations. Each column in **A–J** represents an average of data from the following number of wells with cells: 16 (**A–F**, **H**), 8 (**G, I, J**). Bars are equal to standard deviations. P-values (written in black) are provided for cells transfected with an empty vector and treated with a ligand (e.g. BMP2 or GDF5) compared to cells transfected with a co-receptor of interest (e.g. RGM) and treated with a ligand (e.g. BMP2 or GDF5). P-values (written in blue) are provided for cells transfected with full length RGMB and treated with a ligand (e.g. BMP2 or GDF5) compared to cells transfected with a variant of RGMB (ΔN or one of three mutants) and treated with a ligand (e.g. BMP2 or GDF5). Student's t-test with two-tailed distribution and two-sample unequal variance was used to calculate p-values.

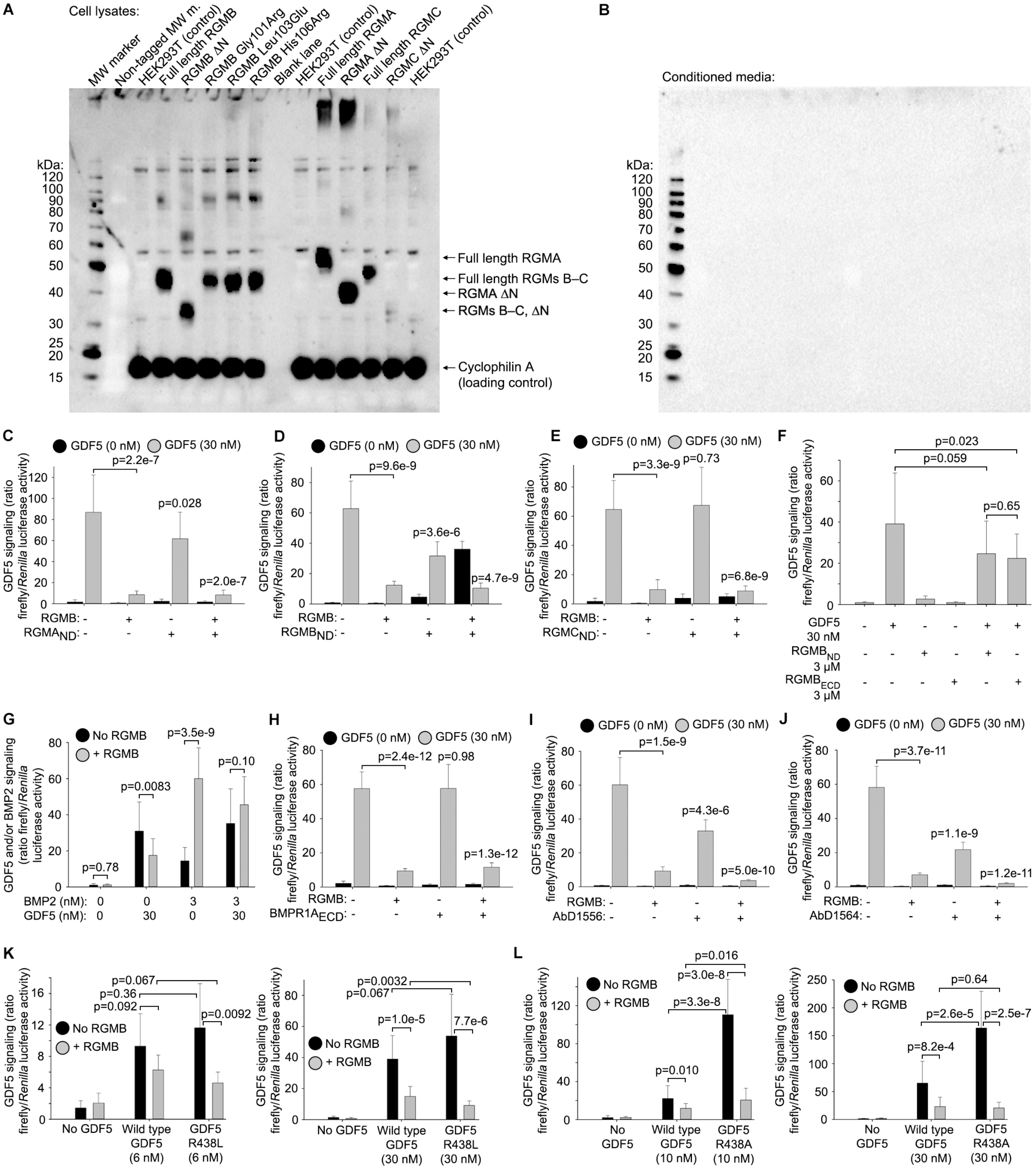
A
Cell lysates:
MW marker
Non-tagged MW m.
HEK293T (control)
Full length RGMB
RGMB ΔN
RGMB Gly101Arg
RGMB Leu103Glu
RGMB His106Arg
Blank lane
HEK293T (control)
Full length RGMA
RGMA ΔN
Full length RGMC
RGMC ΔN
HEK293T (control)
kDa:
120
100
90
80
70
60
50
40
30
25
20
15
← Full length RGMA
← Full length RGMs B–C
← RGMA ΔN
← RGMs B–C, ΔN
← Cyclophilin A
(loading control)
B
Conditioned media:
kDa:
C
GDF5 (0 nM)
GDF5 (30 nM)
p=2.2e-7
p=0.028
p=2.0e-7
GDF5 signaling (ratio firefly/Renilla luciferase activity)
RGMB:
RGMA_ND:
D
p=9.6e-9
p=3.6e-6
p=4.7e-9
RGMB_ND:
E
p=3.3e-9
p=0.73
p=6.8e-9
RGMC_ND:
F
p=0.023
p=0.059
p=0.65
GDF5 30 nM
RGMB_ND 3 μM
RGMB_ECD 3 μM
G
No RGMB
+ RGMB
p=0.78
p=0.0083
p=3.5e-9
p=0.10
GDF5 and/or BMP2 signaling (ratio firefly/Renilla luciferase activity)
BMP2 (nM):
GDF5 (nM):
H
p=2.4e-12
p=0.98
p=1.3e-12
BMPR1A_ECD:
I
p=1.5e-9
p=4.3e-6
p=5.0e-10
AbD1556:
J
p=3.7e-11
p=1.1e-9
p=1.2e-11
AbD1564:
K
p=0.067
p=0.36
p=0.092
p=0.0092
No GDF5
Wild type GDF5 (6 nM)
GDF5 R438L (6 nM)
p=0.0032
p=0.067
p=1.0e-5
7.7e-6
Wild type GDF5 (30 nM)
GDF5 R438L (30 nM)
L
p=0.016
p=3.0e-8
p=3.3e-8
p=0.010
No GDF5
Wild type GDF5 (10 nM)
GDF5 R438A (10 nM)
p=0.64
p=2.6e-5
p=2.5e-7
p=8.2e-4
Wild type GDF5 (30 nM)
GDF5 R438A (30 nM)

Fig. S2. Western blot analysis of RGM constructs expressed in HEK293 cells, GDF5-responsive cellular assay of RGM function in kidney cells (LLC-PK1).

A and **B**. Analysis of expression of RGM constructs containing their GPI-anchor essential for membrane attachment. Anti-His-tag and anti-Cyclophilin A (loading control) western blot of HEK293 cell lysates (**A**) and conditioned media (**B**). Wild type and mutant RGMB constructs were expressed at a similar level. Full-length and N-terminal domain-lacking RGMA constructs were expressed at a similar level as RGMB constructs. Two constructs of RGMC were expressed at a lower level compared to RGMA and RGMB. Samples were reduced with 2-mercaptoethanol and boiled before loading on the gel.

C–J. RGMB–GDF5 signaling assay in the presence of purified N-terminal domain of RGMA ($RGMA_{ND}$) (**C**), $RGMB_{ND}$ (**D**), $RGMC_{ND}$ (**E**), $RGMB_{ND}$ and/or $RGMB_{ECD}$ (**F**). RGMB–GDF5/BMP2 signaling assay showing that GDF5 antagonizes RGMB-mediated potentiation of BMP2 signaling (**G**). RGMB–GDF5 signaling assay in the presence of extracellular domain of BMPR1A ($BMPR1A_{ECD}$) (**H**), the Fab (antigen-binding fragment) of anti-BMPR1A monoclonal antibody AbD1556 (**I**), the Fab of anti-BMPR1A monoclonal antibody AbD1564 (**J**) (7). Full-length, cell surface-attached RGMB inhibited GDF5 signaling in all assays (labeled RGMB and a plus sign in panels **C**–**E** and **H**–**J**). $RGMA_{ND}$ (3 µM, **C**) and $RGMB_{ND}$ (3 µM, **D**) did not inhibit GDF5 (30 nM) signaling as efficiently as full length RGMB, suggesting that the localization of RGMs on the cell surface via GPI-anchor contributes to their inhibitory function. $RGMC_{ND}$ (3 µM, **E**) and the extracellular domain of BMPR1A (3 µM, $BMPR1A_{ECD}$, **H**) did not inhibit GDF5 signaling under similar conditions. Purified $RGMB_{ND}$ (3 µM, **D**) potentiated GDF5 signaling via an unknown mechanism. Two fragments of anti-BMPR1A antibodies (20 µg/ml, ~0.5 µM; (7)) downregulated GDF5 (30 nM) signaling about two-fold (**I** and **J**).

K and **L**. Full length RGMB inhibited signaling by GDF5 variants Arg438Leu (**K**) and Arg438Ala (**L**) that bind to BMP type 1 receptor (BMPR1A) with higher affinity (K_ds 4.2 ± 1.51 nM and 2.0 ± 0.68 nM, respectively, determined previously (8)) compared to wild type GDF5 (K_d 16.2 ± 6.38, determined previously (8)).

P-values (Student's t-test; two-tailed assuming unequal variance) are shown for cells transfected with an empty vector and treated with GDF5 compared to cells transfected (or not) with the full length RGMB and treated with an additional purified protein (N-terminal domains of RGMs, $BMPR1A_{ECD}$ or anti-BMPR1A antibodies) in panels **C**–**E** and **H**–**J**. P-values for indicated datasets are shown in panels **F**, **G**, **K** and **L**. Each column represents the average of data from 16 wells (except the left panel in **K**, 8 wells) with cells in panels **C**–**L**. Capped bars are equal to standard deviations.

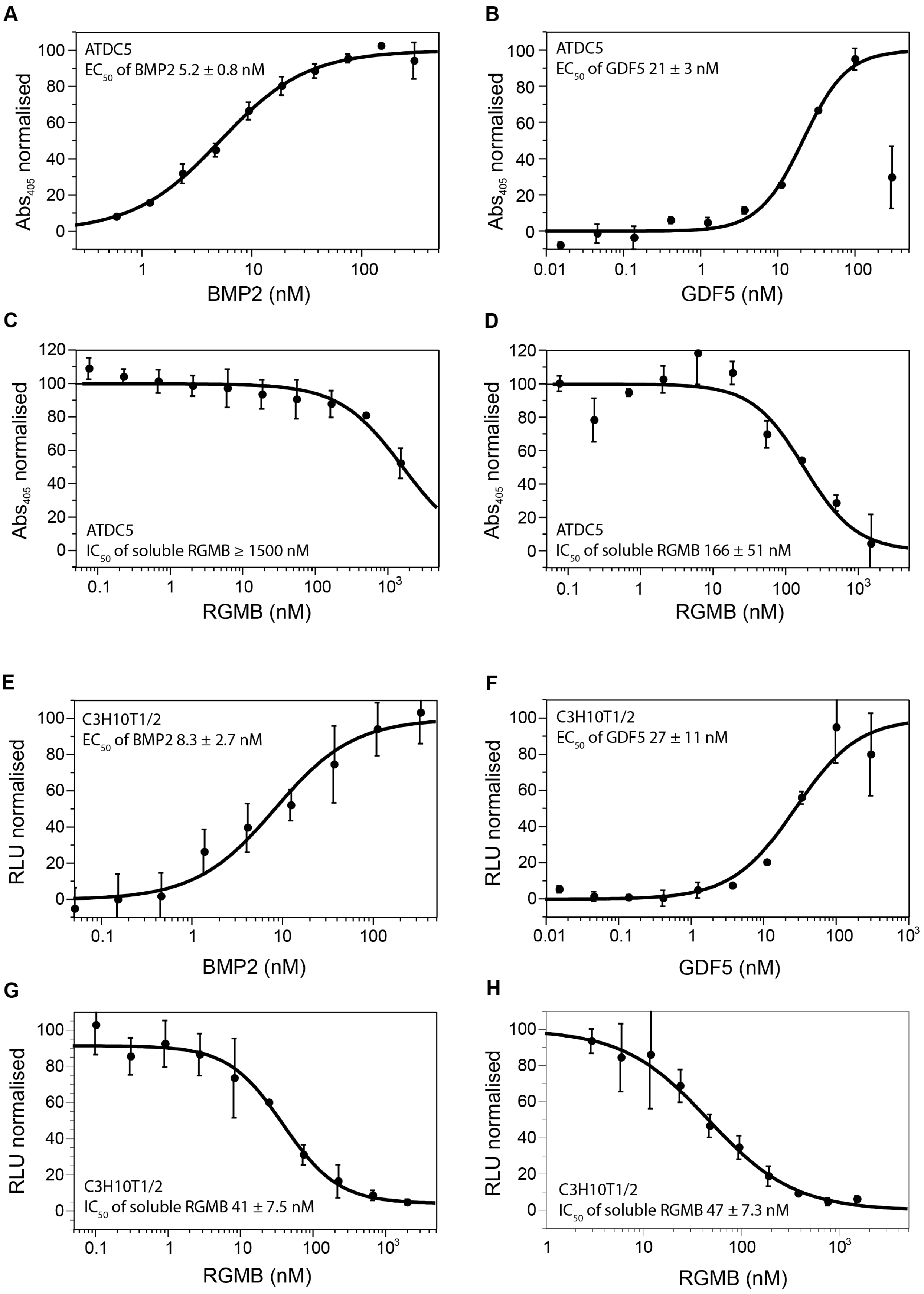
A
ATDC5
EC50 of BMP2 5.2 ± 0.8 nM
Abs405 normalised
BMP2 (nM)
B
ATDC5
EC50 of GDF5 21 ± 3 nM
Abs405 normalised
GDF5 (nM)
C
ATDC5
IC50 of soluble RGMB ≥ 1500 nM
Abs405 normalised
RGMB (nM)
D
ATDC5
IC50 of soluble RGMB 166 ± 51 nM
Abs405 normalised
RGMB (nM)
E
C3H10T1/2
EC50 of BMP2 8.3 ± 2.7 nM
RLU normalised
BMP2 (nM)
F
C3H10T1/2
EC50 of GDF5 27 ± 11 nM
RLU normalised
GDF5 (nM)
G
C3H10T1/2
IC50 of soluble RGMB 41 ± 7.5 nM
RLU normalised
RGMB (nM)
H
C3H10T1/2
IC50 of soluble RGMB 47 ± 7.3 nM
RLU normalised
RGMB (nM)

Fig. S3. Modulation of BMP2- and GDF5-induced expression of alkaline phosphatase in the murine pre-chondrogenic cell line ATDC5 by soluble RGMB (**A–D**) and modulation of BMP2- and GDF5-induced SMAD signaling in the murine mesenchymal stem cell line C3H10T1/2 by soluble RGMB (**E–H**).

Dose-dependent induction of alkaline phosphatase (ALP) expression by BMP2 (**A**) and GDF5 (**B**) in the murine pre-chondrogenic ATDC5 cell line. Each experiment was performed in duplicate on a single micro titer plate. Relative normalized absorbance values are shown. The half-maximal effective concentration (EC_{50}) of BMP2 was 5.2 ± 0.8 nM (**A**) and EC_{50} of GDF5 was 21 ± 3 nM (**B**). **C**, inhibition of BMP2-induced ALP expression by soluble RGMB. Cells were stimulated with 10 nM BMP2 and competition was determined from a log3 dilution of soluble RGMB protein starting with 1.5 μM as the highest concentration. Regression analysis of the dose-dependent inhibition indicates a half-maximal inhibitory concentration (IC_{50}) for RGMB ≥ 1500 nM. Using EC_{50} and IC_{50} values obtained from both assays, the inhibitory constant for RGMB can be derived from the Cheng-Prusoff equation (4) suggesting a $K_i > 500$ nM. **D**, inhibition of GDF5-induced ALP expression by soluble RGMB. Cells were stimulated with 20 nM GDF5 and inhibition of GDF5 signaling by RGMB was determined from a log3 dilution series of soluble RGMB protein with 1.5 μM as the highest concentration. Regression analysis yielded a half-maximal inhibitory concentration (IC_{50}) for RGMB of 166 ± 51 nM. From the EC_{50} and IC_{50} values an inhibitory constant K_i for RGMB of approximately 85 nM is obtained.
Dose-dependent activation of SMAD signaling by BMP2 (**E**) and GDF5 (**F**) in C3H10T1/2 cells. The murine mesenchymal stem cell line C3H10T1/2 stably transfected with a BRE-Luc reporter expression construct (gift of P. ten Dijke, Leiden; (3)) was used to determine the half-maximal effective concentration for SMAD1/5/8 activation. Each experiment was performed in duplicate on a single micro titer plate. The upper panels show dose-dependent SMAD signaling (measured as normalized relative light units (RLU) from the expressed luciferase reporter gene) for BMP2 (**E**) and GDF5 (**F**). The half-maximal effective concentration (EC_{50}) of BMP2 was 8.3 ± 2.7 nM and the EC_{50} of GDF5 was 27 ± 11 nM. For determining the effect of soluble RGMB on BMP2- or GDF5-induced SMAD signaling, C3H10T1/2-BRE cells were stimulated with 10 nM BMP2 or GDF5 in the presence of a log3 or log2 dilution series of soluble RGMB starting with a highest concentration of 2000 nM (BMP2) and 1500 nM (GDF5). Stimulation with 10 nM BMP2 and GDF5 without addition of soluble RGMB was used as a control, as well as a log3 or log2 dilution of soluble RGMB without stimulation by BMP2 or GDF5. **G**, a half-maximal inhibitory concentration (IC_{50}) of 41 ± 7.5 nM was determined for the RGMB-mediated inhibition of BMP2-induced SMAD signaling, while for RGMB-mediated inhibition of GDF5-induced SMAD signaling (**H**) an IC_{50} of about 47 ± 7.3 nM was observed.

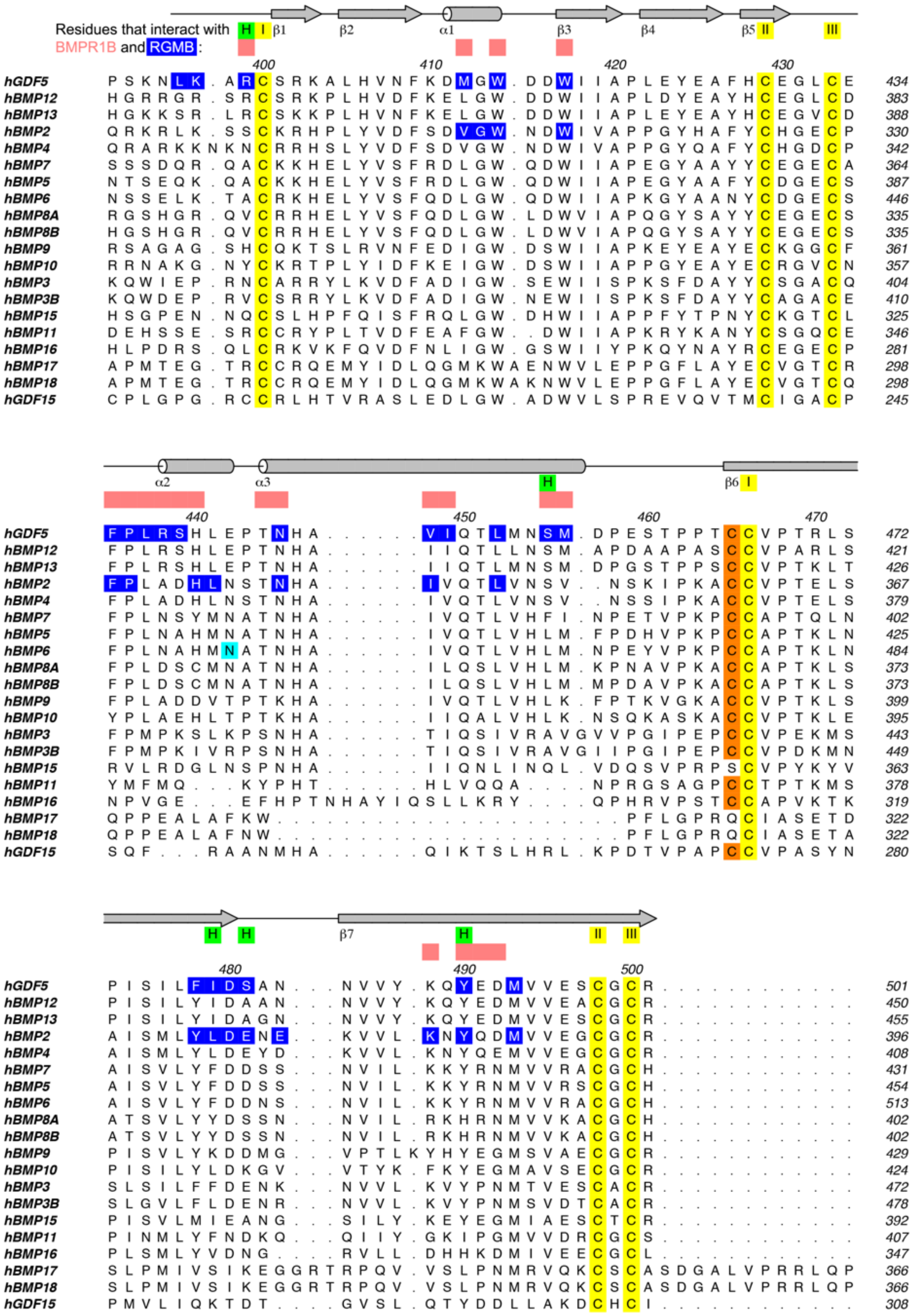
Residues that interact with H I
BMPR1B and RGMB:
β1 β2 α1 β3 β4 β5 II III
400 410 420 430
hGDF5 PSKNLK.ARCSRKALHVNFKDMGW.DDWIIAPLEYEAFHCEGLCE 434
hBMP12 HGRRGR.SRCSRKPLHVDFKELGW.DDWIIAPLDYEAYHCEGLCD 383
hBMP13 HGKKSR.LRCSKKPLHVNFKELGW.DDWIIAPLEYEAYHCEGVCD 388
hBMP2 QRKRLK.SSCKRHPLYVDFSDVGW.NDWIVAPPGYHAFYCHGECP 330
hBMP4 QRARKKNKNCRRHSLYVDFSDVGW.NDWIVAPPGYQAFYCHGDCP 342
hBMP7 SSSDQR.QACKKHELYVSFRDLGW.QDWIIAPEGYAAYYCEGECA 364
hBMP5 NTSEQK.QACKKHELYVSFRDLGW.QDWIIAPEGYAAFYCDGECS 387
hBMP6 NSSELK.TACRKHELYVSFQDLGW.QDWIIAPKGYAANYCDGECS 446
hBMP8A RGSHGR.QVCRRHELYVSFQDLGW.LDWVIAPQGYSAYYCEGECS 335
hBMP8B HGSHGR.QVCRRHELYVSFQDLGW.LDWVIAPQGYSAYYCEGECS 335
hBMP9 RSAGAG.SHCQKTSLRVNFEDIGW.DSWIIAPKEYEAYECKGGCF 361
hBMP10 RRNAKG.NYCKRTPLYIDFKEIGW.DSWIIAPPGYEAYECRGVCN 357
hBMP3 KQWIEP.RNCARRYLKVDFADIGW.SEWIISPKSFDAYYCSGACQ 404
hBMP3B KQWDEP.RVCSRRYLKVDFADIGW.NEWIISPKSFDAYYCAGACE 410
hBMP15 HSGPEN.NQCSLHPFQISFRQLGW.DHWIIAPPFYTPNYCKGTCL 325
hBMP11 DEHSSE.SRCCRYPLTVDFEAFGW..DWIIAPKRYKANYCSGQCE 346
hBMP16 HLPDRS.QLCRKVKFQVDFNLIGW.GSWIIYPKQYNAYRCEGECP 281
hBMP17 APMTEG.TRCCRQEMYIDLQGMKWAENWVLEPPGFLAYECVGTCR 298
hBMP18 APMTEG.TRCCRQEMYIDLQGMKWAKNWVLEPPGFLAYECVGTCQ 298
hGDF15 CPLGPG.RCCRLHTVRASLEDLGW.ADWVLSPREVQVTMCIGACP 245
α2 α3 H β6 I
440 450 460 470
hGDF5 FPLRSHLEPTNHA......VIQTLMNSM.DPESTPPTCCVPTRLS 472
hBMP12 FPLRSHLEPTNHA......IIQTLLNSM.APDAAPASCCVPARLS 421
hBMP13 FPLRSHLEPTNHA......IIQTLMNSM.DPGSTPPSCCVPTKLT 426
hBMP2 FPLADHLNSTNHA......IVQTLVNSV..NSKIPKACCVPTELS 367
hBMP4 FPLADHLNSTNHA......IVQTLVNSV..NSSIPKACCVPTELS 379
hBMP7 FPLNSYMNATNHA......IVQTLVHFI.NPETVPKPCCAPTQLN 402
hBMP5 FPLNAHMNATNHA......IVQTLVHLM.FPDHVPKPCCAPTKLN 425
hBMP6 FPLNAHMNATNHA......IVQTLVHLM.NPEYVPKPCCAPTKLN 484
hBMP8A FPLDSCMNATNHA......ILQSLVHLM.KPNAVPKACCAPTKLS 373
hBMP8B FPLDSCMNATNHA......ILQSLVHLM.MPDAVPKACCAPTKLS 373
hBMP9 FPLADDVTPTKHA......IVQTLVHLK.FPTKVGKACCVPTKLS 399
hBMP10 YPLAEHLTPTKHA......IIQALVHLK.NSQKASKACCVPTKLE 395
hBMP3 FPMPKSLKPSNHA......TIQSIVRAVGVVPGIPEPCCVPEKMS 443
hBMP3B FPMPKIVRPSNHA......TIQSIVRAVGIIPGIPEPCCVPDKMN 449
hBMP15 RVLRDGLNSPNHA......IIQNLINQL.VDQSVPRPSCVPYKYV 363
hBMP11 YMFMQ...KYPHT......HLVQQA....NPRGSAGPCCTPTKMS 378
hBMP16 NPVGE...EFHPTNHAYIQSLLKRY....QPHRVPSTCCAPVKTK 319
hBMP17 QPPEALAFKW.....................PFLGPRQCIASETD 322
hBMP18 QPPEALAFNW.....................PFLGPRQCIASETA 322
hGDF15 SQF...RAANMHA......QIKTSLHRL.KPDTVPAPCCVPASYN 280
H H β7 H II III
480 490 500
hGDF5 PISILFIDSAN...NVVY.KQYEDMVVESCGCR............ 501
hBMP12 PISILYIDAAN...NVVY.KQYEDMVVEACGCR............ 450
hBMP13 PISILYIDAGN...NVVY.KQYEDMVVESCGCR............ 455
hBMP2 AISMLYLDENE...KVVL.KNYQDMVVEGCGCR............ 396
hBMP4 AISMLYLDEYD...KVVL.KNYQEMVVEGCGCR............ 408
hBMP7 AISVLYFDDSS...NVIL.KKYRNMVVRACGCH............ 431
hBMP5 AISVLYFDDSS...NVIL.KKYRNMVVRSCGCH............ 454
hBMP6 AISVLYFDDNS...NVIL.KKYRNMVVRACGCH............ 513
hBMP8A ATSVLYYDSSN...NVIL.RKHRNMVVKACGCH............ 402
hBMP8B ATSVLYYDSSN...NVIL.RKHRNMVVKACGCH............ 402
hBMP9 PISVLYKDDMG...VPTLKYHYEGMSVAECGCR............ 429
hBMP10 PISILYLDKGV...VTYK.FKYEGMAVSECGCR............ 424
hBMP3 SLSILFFDENK...NVVL.KVYPNMTVESCACR............ 472
hBMP3B SLGVLFLDENR...NVVL.KVYPNMSVDTCACR............ 478
hBMP15 PISVLMIEANG...SILY.KEYEGMIAESCTCR............ 392
hBMP11 PINMLYFNDKQ...QIIY.GKIPGMVVDRCGCS............ 407
hBMP16 PLSMLYVDNG....RVLL.DHHKDMIVEECGCL............ 347
hBMP17 SLPMIVSIKEGGRTRPQV.VSLPNMRVQKCSCASDGALVPRRLQP 366
hBMP18 SLPMIVSIKEGGRTRPQV.VSLPNMRVQKCSCASDGALVPRRLQP 366
hGDF15 PMVLIQKTDT....GVSL.QTYDDLLAKDCHCI............ 308

Fig. S4. Structure-based alignment of amino acid sequences of GDF and BMP family members.

Amino acid sequences are taken from the UniProt database (9) (h, human; entry code): hBMP2 (P12643), hBMP3 (also known as Osteogenin, P12645), hBMP3B (GDF10; P55107), hBMP4 (BMP2B, P12644), hBMP5 (P22003), hBMP6 (VGR1, P22004), hBMP7 (OP1, P18075), hBMP8A (Q7Z5Y6), hBMP8B (OP2, P34820), hBMP9 (GDF2, Q9UK05), hBMP10 (O95393), hBMP11 (GDF11, O95390), hBMP12 (GDF7, Q7Z4P5), hBMP13 (GDF6, CDMP2, Q6KF10), GDF5 (BMP14, CDMP1, P43026), hBMP15 (GDF9B, O95972), hBMP16 (Nodal, Q96S42), hBMP17 (LEFTY1, O75610), hBMP18 (LEFTY2, O00292), hGDF15 (Q99988). Human BMP1 (also known as Procollagen C-peptidase, P13497) does not belong to the TGF-β family. Sequences were aligned using PROMALS3D (10) using constraints from GDF5 (from the high resolution RGMB–GDF5 complex presented here), BMP2 (Protein Data Bank ID 1REW; (11)), BMP3 (2QCQ; (12)), BMP6 (2R52; (13)), BMP7 (1BMP; (14)), BMP9 (4MPL; (15)), BMP11/GDF11 (5E4G; (16)), and GDF15 (5VZ4; (17)). Sequences were formatted using ALINE (18). Secondary structure elements of GDF5 are shown above the alignment and were assigned using PDBsum (19), and correspond to **Fig. 1F**. Residues at the RGMB–GDF5 (this study, 1.7 Å structure) and RGMB–BMP2 (PDB ID 4UHZ; (5)) interfaces were identified using PDBsum (19) and are highlighted in blue. Five residues that form hydrogen bonds at the RGMB–GDF5 interface are marked with H. Six cysteines that form intramolecular disulfide bonds (numbered I–III) are highlighted in yellow, cysteines that form intermolecular disulfide bonds are highlighted in orange. The N-linked glycosylation site on BMP6 is highlighted in cyan (13). Residues of GDF5 with the largest buried surface area (BSA ≥ 70 $Å^2$; $Å^2$) in the RGMB–GDF5 complex: R399 (70), W417 (77), F435 (93), M456 (72), S481 (74). BSA was calculated using PISA (20). Residues of GDF5 that interact with BMPR1B are marked with pink squares (PDB ID 3EVS; (8)).

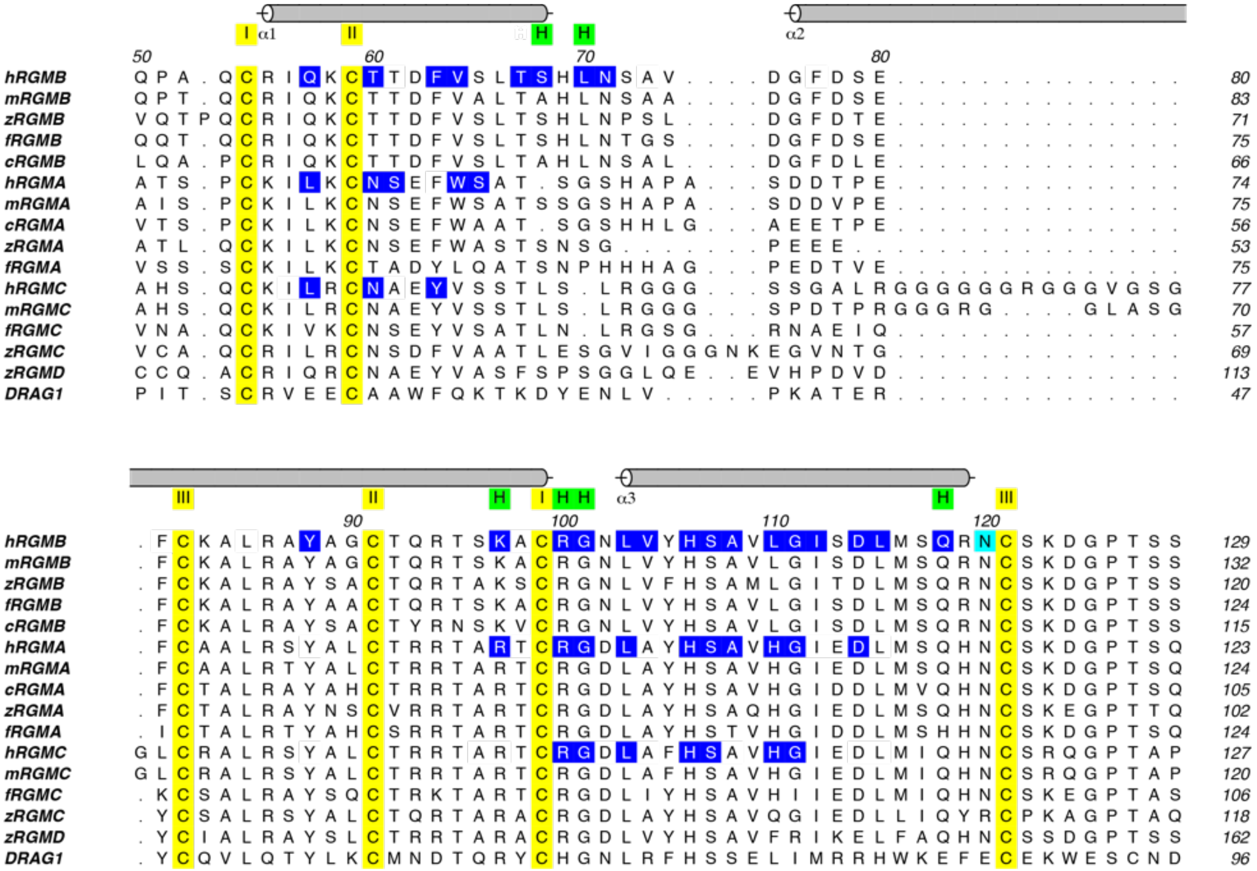

Fig. S5. Structure-based alignment of amino acid sequences of RGM family members.

Amino acid sequences were taken from the UniProt database (9) (h, human; m, mouse; c, chick; f, frog; z, zebrafish; entry code: hRGMA (Q96B86), hRGMB (Q6NW40), hRGMC (Q6ZVN8), mRGMA (Q6PCX7), mRGMB (Q7TQ33), mRGMC (Q7TQ32), cRGMA (Q8JG54), cRGMB (F1NNI0), xRGMA (Q6P8A0), xRGMB (Q6P8A4), xRGMC (A0A1L8FBY8), zRGMA (Q6IYC9), zRGMB (Q6IYD1), zRGMC (Q1JQE7), zRGMD (E9QCG3), DRAG1 (G5EDE5) from *Caenorhabditis elegans.* Sequences were formatted using ALINE (18). Secondary structure elements of RGMB are shown above the alignment and were assigned using PDBsum (19), and correspond to **Fig. 1F–I**. Residues at the RGM–GDF5 interfaces were identified using PDBsum (19) and are highlighted in blue. Six residues that form hydrogen bonds are marked with H. Six cysteines that form intramolecular disulfide bonds (numbered I–III) are highlighted in yellow. The N-linked glycosylation site on Asn120 of RGMB is highlighted in cyan. Residues of RGMA with the largest buried surface area in the RGMA–GDF5 complex (BSA ≥ 70 Å^2; Å^2): W58 (149), R94 (105), L97 (144), S101 (71), H104 (91). Residues of RGMB with the largest BSA in the RGMB–GDF5 complex (BSA ≥ 70 Å^2; Å^2): R100 (125), L103 (138), L110 (97). Residues of RGMC with the largest BSA the RGMC–GDF5 complex (BSA ≥ 70 Å^2; Å^2): L40 (103), R98 (91), L101 (157), S105 (71), H108 (85). Buried surface area (BSA) was calculated using PISA (19).

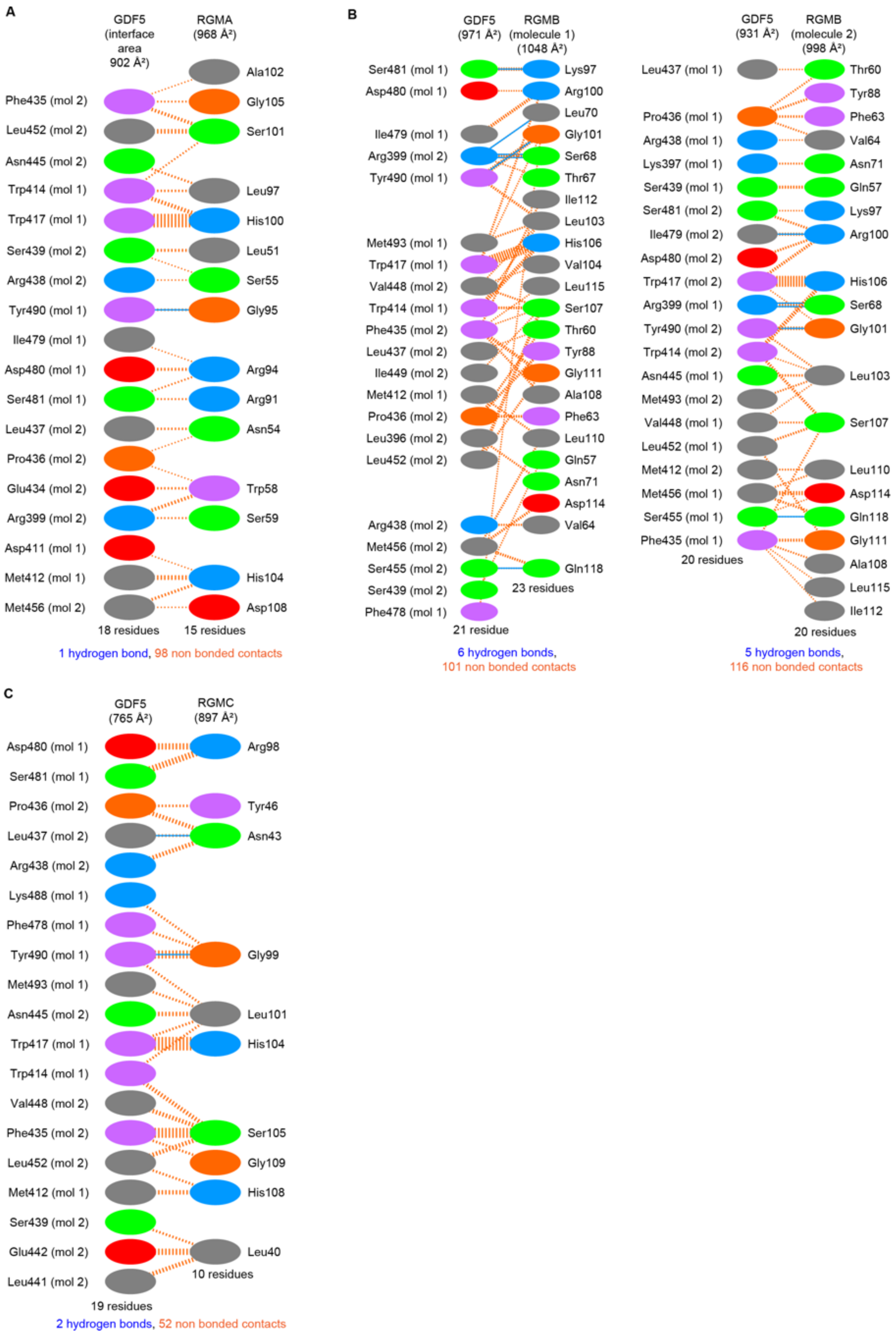
A
GDF5 (interface area 902 Å²)
RGMA (968 Å²)
Phe435 (mol 2)
Leu452 (mol 2)
Asn445 (mol 2)
Trp414 (mol 1)
Trp417 (mol 1)
Ser439 (mol 2)
Arg438 (mol 2)
Tyr490 (mol 1)
Ile479 (mol 1)
Asp480 (mol 1)
Ser481 (mol 1)
Leu437 (mol 2)
Pro436 (mol 2)
Glu434 (mol 2)
Arg399 (mol 2)
Asp411 (mol 1)
Met412 (mol 1)
Met456 (mol 2)
Ala102
Gly105
Ser101
Leu97
His100
Leu51
Ser55
Gly95
Arg94
Arg91
Asn54
Trp58
Ser59
His104
Asp108
18 residues
15 residues
1 hydrogen bond, 98 non bonded contacts
B
GDF5 (971 Å²)
RGMB (molecule 1) (1048 Å²)
Ser481 (mol 1)
Asp480 (mol 1)
Ile479 (mol 1)
Arg399 (mol 2)
Tyr490 (mol 1)
Met493 (mol 1)
Trp417 (mol 1)
Val448 (mol 2)
Trp414 (mol 1)
Phe435 (mol 2)
Leu437 (mol 2)
Ile449 (mol 2)
Met412 (mol 1)
Pro436 (mol 2)
Leu396 (mol 2)
Leu452 (mol 2)
Arg438 (mol 2)
Met456 (mol 2)
Ser455 (mol 2)
Ser439 (mol 2)
Phe478 (mol 1)
Lys97
Arg100
Leu70
Gly101
Ser68
Thr67
Ile112
Leu103
His106
Val104
Leu115
Ser107
Thr60
Tyr88
Gly111
Ala108
Phe63
Leu110
Gln57
Asn71
Asp114
Val64
Gln118
21 residue
23 residues
6 hydrogen bonds, 101 non bonded contacts
GDF5 (931 Å²)
RGMB (molecule 2) (998 Å²)
Leu437 (mol 1)
Pro436 (mol 1)
Arg438 (mol 1)
Lys397 (mol 1)
Ser439 (mol 1)
Ser481 (mol 2)
Ile479 (mol 2)
Asp480 (mol 2)
Trp417 (mol 2)
Arg399 (mol 1)
Tyr490 (mol 2)
Trp414 (mol 2)
Asn445 (mol 1)
Met493 (mol 2)
Val448 (mol 1)
Leu452 (mol 1)
Met412 (mol 2)
Met456 (mol 1)
Ser455 (mol 1)
Phe435 (mol 1)
Thr60
Tyr88
Phe63
Val64
Asn71
Gln57
Lys97
Arg100
His106
Ser68
Gly101
Leu103
Ser107
Leu110
Asp114
Gln118
Gly111
Ala108
Leu115
Ile112
20 residues
20 residues
5 hydrogen bonds, 116 non bonded contacts
C
GDF5 (765 Å²)
RGMC (897 Å²)
Asp480 (mol 1)
Ser481 (mol 1)
Pro436 (mol 2)
Leu437 (mol 2)
Arg438 (mol 2)
Lys488 (mol 1)
Phe478 (mol 1)
Tyr490 (mol 1)
Met493 (mol 1)
Asn445 (mol 2)
Trp417 (mol 1)
Trp414 (mol 1)
Val448 (mol 2)
Phe435 (mol 2)
Leu452 (mol 2)
Met412 (mol 1)
Ser439 (mol 2)
Glu442 (mol 2)
Leu441 (mol 2)
Arg98
Tyr46
Asn43
Gly99
Leu101
His104
Ser105
Gly109
His108
Leu40
19 residues
10 residues
2 hydrogen bonds, 52 non bonded contacts

Fig. S6. Interfaces between RGMs and GDF5 in the binary RGM–GDF5 complexes.

Residues at the interface between GDF5 and RGMA (**A**), RGMB (**B**), and RGMC (**C**) were analyzed and surface interface areas were calculated using PDBsum (19). The number of hydrogen bond lines (blue) between any two residues indicates the number of potential hydrogen bonds between them (19). For non-bonded contacts (van der Waals interactions), the width of the striped line (orange) is proportional to the number of atomic contacts (19), e.g. note extensive atomic contacts between Trp417 of GDF5 and histidines from RGMs. Residue colors: positive (H, K, R), blue; negative (D, E), red; neutral (S, T, N, Q), green; aliphatic (A, V, L, I, M), gray; aromatic (F, Y, W), violet; orange (proline and glycine). RGMA–GDF5 and RGMC–GDF5 form a 1:1 mol:mol complex in the crystallographic asymmetric unit, where two disulfide linked protomers of GDF5 dimer are related by a crystallographic two-fold axis, thus identical interactions between GDF5 and the two RGM molecules in the biological unit are observed. In contrast, RGMB–GDF5 forms a 2:2 mol:mol complex in the crystallographic asymmetric unit, and the interactions between two distinct RGMB molecules and the GDF5 dimer were analyzed (**B**).

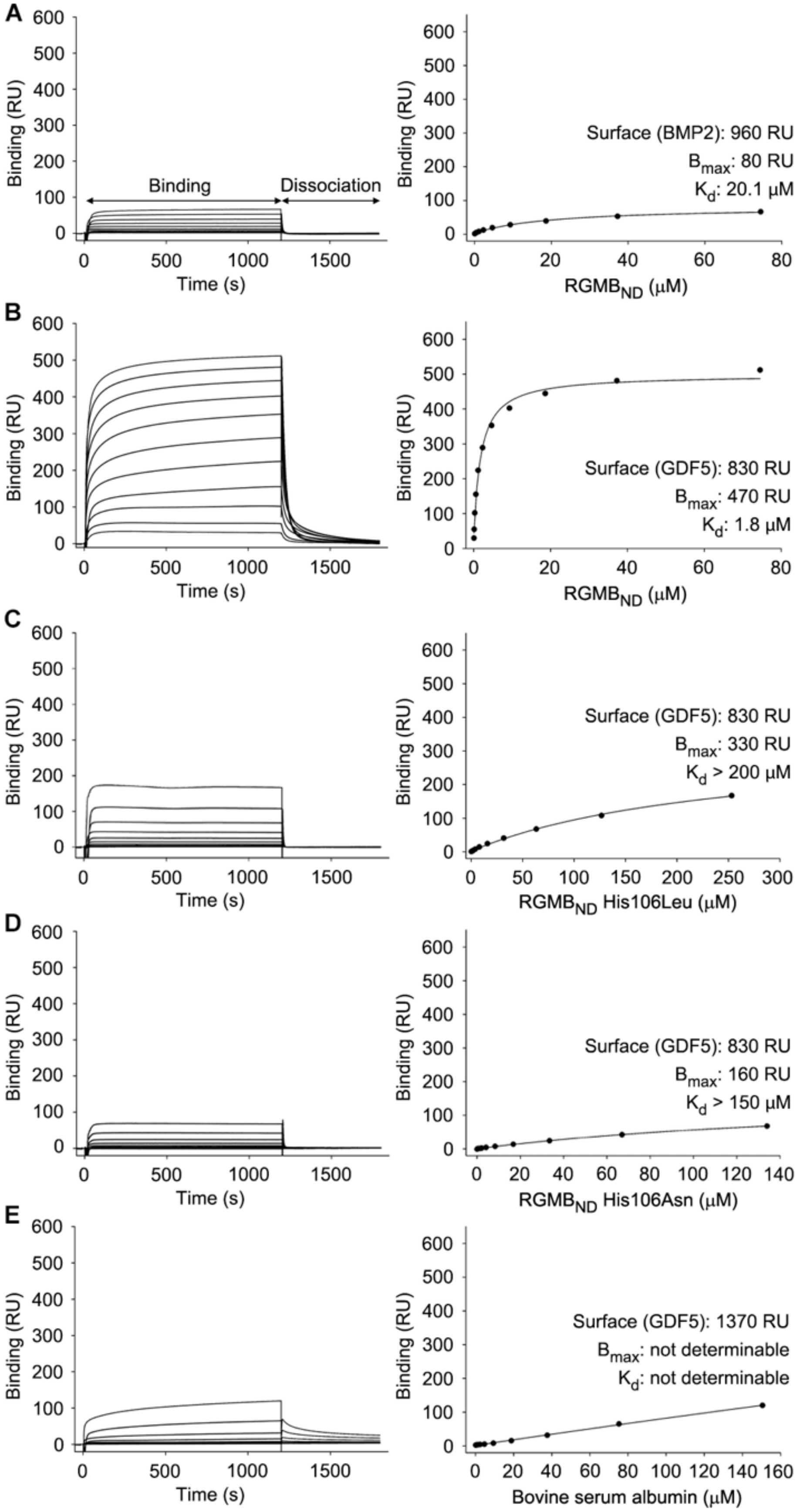
A
Binding (RU)
Binding
Dissociation
Time (s)
Surface (BMP2): 960 RU
B_max: 80 RU
K_d: 20.1 μM
RGMB_ND (μM)
B
Binding (RU)
Time (s)
Surface (GDF5): 830 RU
B_max: 470 RU
K_d: 1.8 μM
RGMB_ND (μM)
C
Binding (RU)
Time (s)
Surface (GDF5): 830 RU
B_max: 330 RU
K_d > 200 μM
RGMB_ND His106Leu (μM)
D
Binding (RU)
Time (s)
Surface (GDF5): 830 RU
B_max: 160 RU
K_d > 150 μM
RGMB_ND His106Asn (μM)
E
Binding (RU)
Time (s)
Surface (GDF5): 1370 RU
B_max: not determinable
K_d: not determinable
Bovine serum albumin (μM)

Fig. S7. Surface plasmon resonance (SPR)-based equilibrium binding experiments between RGMs and GDF5 in the presence of high concentrations of Tween 20 (0.05%, v/v).

A–E, SPR-based equilibrium binding experiments showing direct interactions between $RGMB_{ND}$ and BMP2 (**A**), $RGMB_{ND}$ and GDF5 (**B**), $RGMB_{ND}$ His106Leu and GDF5 (**C**), $RGMB_{ND}$ His106Asn and GDF5 (**D**), and very weak interactions between $RGMB_{ND}$ and bovine serum albumin (BSA, **E**). $RGMB_{ND}$ interacts more weakly with BMP2 (**A**) compared to GDF5 (**B**) under identical conditions. Mutations of RGMB residues that eliminate π–π stacking between RGMB His106 and GDF5 Trp417 (**Fig. 1I**) attenuate the $RGMB_{ND}$–GDF5 interaction. SPR sensorgrams and corresponding isotherms are shown. RU, response units. B_{max}, maximum response at saturating concentration of analyte (RGMB or BSA). Experiments were performed in the following SPR running buffer: 0.5 M NaCl, 20 mM HEPES pH 7.4, 0.05% (v/v) Tween 20.

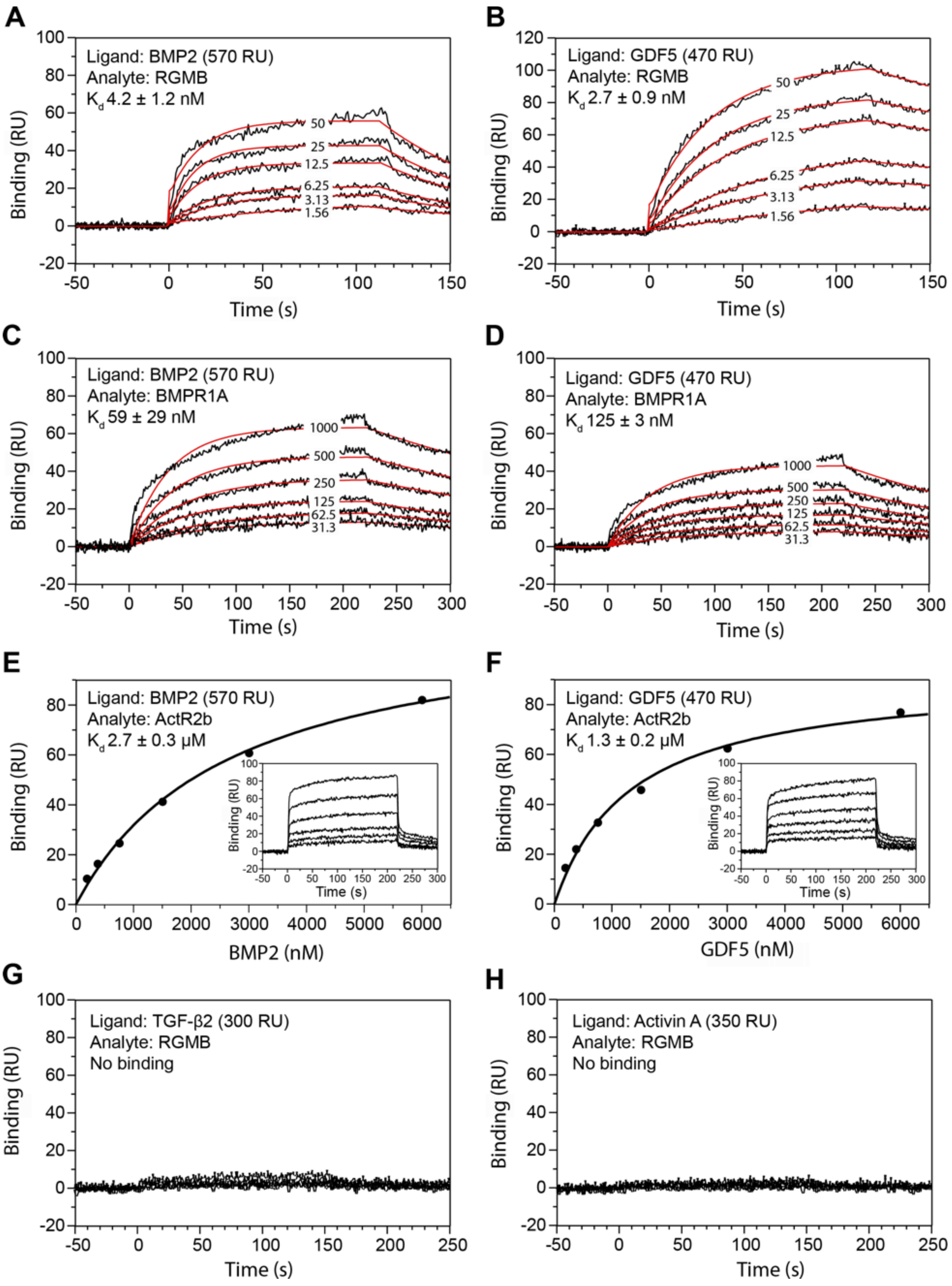
A
Ligand: BMP2 (570 RU)
Analyte: RGMB
K_d 4.2 ± 1.2 nM
50
25
12.5
6.25
3.13
1.56
Binding (RU)
Time (s)
B
Ligand: GDF5 (470 RU)
Analyte: RGMB
K_d 2.7 ± 0.9 nM
50
25
12.5
6.25
3.13
1.56
Binding (RU)
Time (s)
C
Ligand: BMP2 (570 RU)
Analyte: BMPR1A
K_d 59 ± 29 nM
1000
500
250
125
62.5
31.3
Binding (RU)
Time (s)
D
Ligand: GDF5 (470 RU)
Analyte: BMPR1A
K_d 125 ± 3 nM
1000
500
250
125
62.5
31.3
Binding (RU)
Time (s)
E
Ligand: BMP2 (570 RU)
Analyte: ActR2b
K_d 2.7 ± 0.3 μM
Binding (RU)
Time (s)
BMP2 (nM)
F
Ligand: GDF5 (470 RU)
Analyte: ActR2b
K_d 1.3 ± 0.2 μM
Binding (RU)
Time (s)
GDF5 (nM)
G
Ligand: TGF-β2 (300 RU)
Analyte: RGMB
No binding
Binding (RU)
Time (s)
H
Ligand: Activin A (350 RU)
Analyte: RGMB
No binding
Binding (RU)
Time (s)

Fig. S8. Surface plasmon resonance (SPR)-based binding experiments between BMP2, GDF5 and their binding partners in the presence of lower concentration of Tween 20 (0.005%, v/v).

In vitro binding analysis of the interaction of RGMB with BMP2 (**A**) and GDF5 (**B**). For SPR measurements, BMP2 and GDF5 protein were immobilized on a GLC sensor chip using primary amine coupling at a density of about 570 and 470 RU respectively. RGMB protein was dissolved in 20 mM HEPES pH 7.4, 500 mM NaCl, 3.4 mM EDTA, 0.005% (v/v) Tween 20 (SPR running buffer) and was perfused as analyte at six concentrations (50 nM to 1.56 nM) at a flow rate of 150 µl/min. Association was monitored for 160 s, dissociation was initiated by perfusing the biosensor with running buffer. Due to fast rebinding, which severely hampered regression analysis of the sensorgrams, dissociation was monitored only for 35 s. Despite the short dissociation phase, regression analysis of the sensorgram had to be performed with fitting parameters treated locally. Equilibrium binding constants were calculated from the equation $K_d = k_{off}/k_{on}$. SPR binding data were acquired from two independent experiments. Using this setup, RGMB bound BMP2 with a K_d of 4.2 ± 1.2 nM, the association rate constant k_{on} was $5.3 \pm 2 \times 10^6$ $M^{-1}s^{-1}$ and the dissociation rate constant k_{off} was $11 \pm 3 \times 10^{-3}$ s^{-1}. Full-length RGMB bound GDF5 with a K_d of 2.7 ± 0.9 nM, association and dissociation rate constants were $1.8 \pm 0.2 \times 10^6$ $M^{-1}s^{-1}$ and $2.5 \pm 0.5 \times 10^{-3}$ s^{-1} respectively. **C** and **D**, as a control, binding parameters of the BMP type-1 receptor BMPR1A was measured. Purified BMPR1A ectodomain protein (residues Gln24 to Arg152) was perfused as analyte in six concentrations ranging from 1000 to 31.25 nM at a flow rate of 100 µl/min. Association was monitored for 220 s, dissociation data were acquired for 80 s. Under these conditions an equilibrium binding constant K_d of 59 ± 29 nM was calculated for binding of BMPR1A to BMP2 (**C**) ($k_{on} = 1.1 \pm 0.1 \times 10^5$ $M^{-1}s^{-1}$, $k_{off} = 2.7 \pm 1 \times 10^{-3}$ s^{-1}) and of 125 ± 3 nM for binding of BMPR1A to GDF5 (**D**) ($k_{on} = 0.9 \pm 0.02 \times 10^5$ $M^{-1}s^{-1}$, $k_{off} = 4.8 \pm 0.03 \times 10^{-3}$ s^{-1}). **E** and **F**, as a control, binding of BMP2 and GDF5 to murine activin type-2 receptor ActR2b was measured. Purified soluble ectodomain protein ActR2b (residues Gly20 to Glu127) was perfused at six concentrations ranging from 6 µM to 187.5 nM at a flow rate of 100 µl/min. Due to fast association/fast dissociation binding kinetics, binding affinities were calculated from the dose-dependency of equilibrium binding and fitted with a one-site binding model. Binding affinity of ActR2b to BMP2 was calculated to 2.7 ± 0.3 µM and a K_d value of 1.3 ± 0.2 µM was obtained for binding of ActR2b to GDF5. **G** and **H**, as a control, binding of RGMB to the TGF-β ligands TGF-β2 and activin A was analyzed. About 300 RU of human TGF-β2 and about 350 RU of human activin A were immobilized on a GLC sensor chip using primary amine coupling. Purified RGMB protein was perfused as analyte at a flow rate of 100 µl/min using six different concentrations (50 to 1.56 nM). Association was monitored for 160 s, dissociation was observed for 140 s. No binding of RGMB protein was observed under these conditions confirming the specificity of RGM proteins for a subset of BMP ligands.

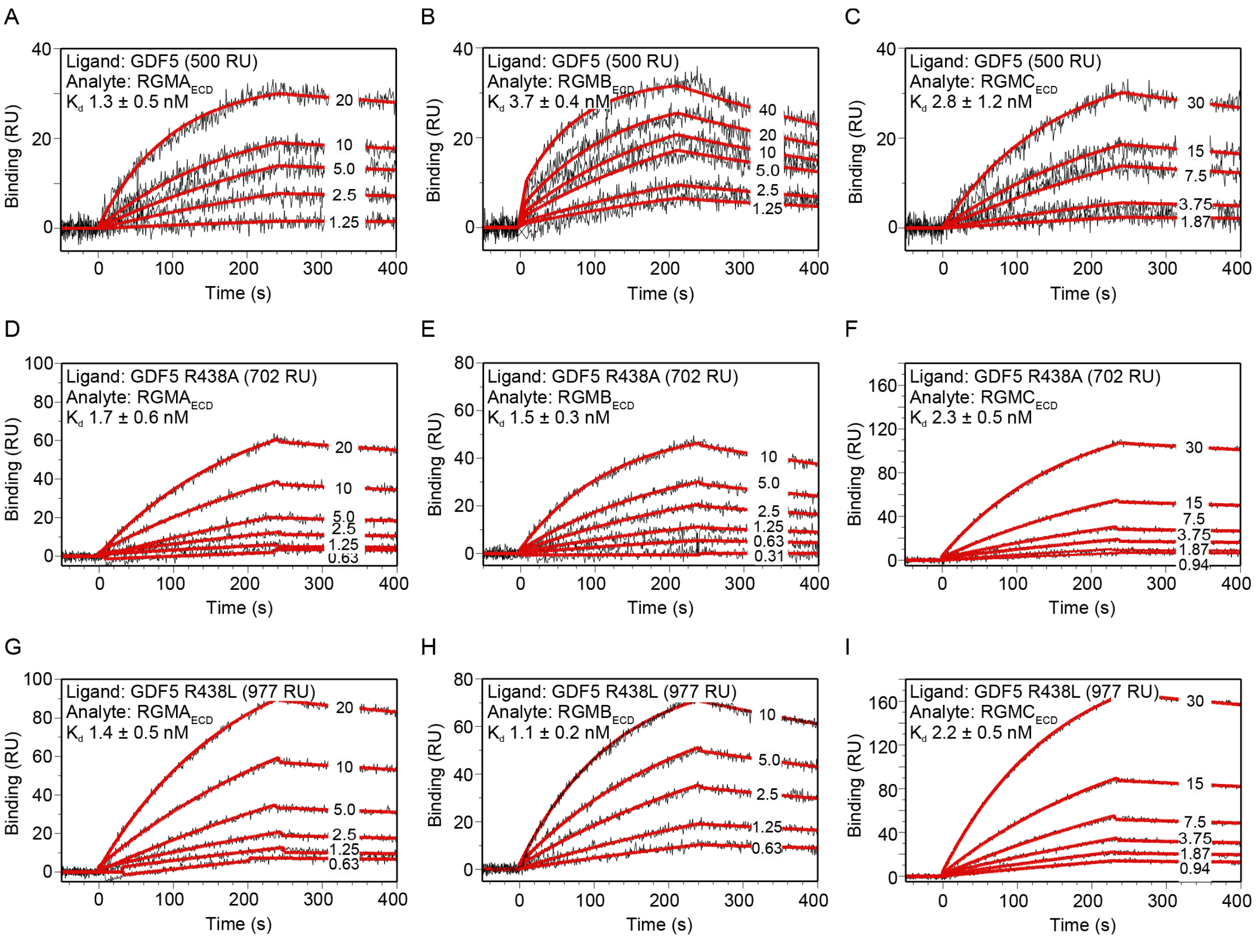
A
Ligand: GDF5 (500 RU)
Analyte: RGMA$_{ECD}$
K_d 1.3 ± 0.5 nM
B
Ligand: GDF5 (500 RU)
Analyte: RGMB$_{ECD}$
K_d 3.7 ± 0.4 nM
C
Ligand: GDF5 (500 RU)
Analyte: RGMC$_{ECD}$
K_d 2.8 ± 1.2 nM
D
Ligand: GDF5 R438A (702 RU)
Analyte: RGMA$_{ECD}$
K_d 1.7 ± 0.6 nM
E
Ligand: GDF5 R438A (702 RU)
Analyte: RGMB$_{ECD}$
K_d 1.5 ± 0.3 nM
F
Ligand: GDF5 R438A (702 RU)
Analyte: RGMC$_{ECD}$
K_d 2.3 ± 0.5 nM
G
Ligand: GDF5 R438L (977 RU)
Analyte: RGMA$_{ECD}$
K_d 1.4 ± 0.5 nM
H
Ligand: GDF5 R438L (977 RU)
Analyte: RGMB$_{ECD}$
K_d 1.1 ± 0.2 nM
I
Ligand: GDF5 R438L (977 RU)
Analyte: RGMC$_{ECD}$
K_d 2.2 ± 0.5 nM
Binding (RU)
Time (s)

Fig. S9. Surface plasmon resonance (SPR)-based binding experiments between the full-length extracellular domains of RGMs A, B, C and GDF5 variants with enhanced affinity to type-1 receptor.

Binding of RGM proteins (RGMA, B and C) to GDF5 variants with enhanced type-1 receptor affinity was analyzed. GDF5 Arg438Ala (middle panels **D–F**) and Arg438Leu (lower panels **G–I**) were shown to bind the type-1 receptor BMPR1A with affinities similar or identical to BMP2 (21, 22) and are thus considered to functionally act as BMP2 mimics (21, 23). While binding of these GDF5 variants to type-1 and type-2 receptors were analyzed in the past, interactions of these GDF5 proteins with RGMs have not been investigated yet. For SPR measurements, the GDF5 proteins were immobilized on a GLC sensor chip using primary amine coupling at a density of about 500 (wild type GDF5), 702 (GDF5 Arg438Ala) and 977 (GDF5 Arg438Leu) RU respectively. Binding of RGMA, B and C to wild type GDF5 was determined for comparison (upper panels **A–C**). For this analysis RGM_{ECD} proteins were derived from transient expression in FreeStyle 293 cell culture (see above). RGM proteins were dissolved in 20 mM HEPES pH 7.4, 500 mM NaCl, 3.4 mM EDTA, 0.005% (v/v) Tween 20 (SPR running buffer) and used as analytes at six concentrations (log2 dilution series starting either at 40, 30, 20 or 10 nM). The highest concentrations were adopted to yield sensorgrams with a sufficiently high signal-to-noise ratio for the lowest concentration in the dilution series, concentrations used are indicated in the individual panels. All SPR experiments were performed using a flow rate of 100 µl/min. Association was monitored for 240 s, dissociation was initiated by perfusing the biosensor with running buffer and monitored for 120 s. To remove bulk face effects and non-specific binding to the chip sensor matrix, binding of the analyte to a non-modified flow channel was subtracted from the raw sensorgram. Regression analyses were performed with fitting parameters for k_{on} (association rate constant) and k_{off} (dissociation rate constant) treated globally. Equilibrium binding constants were calculated from the equation $K_d = k_{off}/k_{on}$. SPR binding data were acquired from two independent experiments. Binding data for equilibrium binding constant, association and dissociation rate constants are indicated in Table S1.

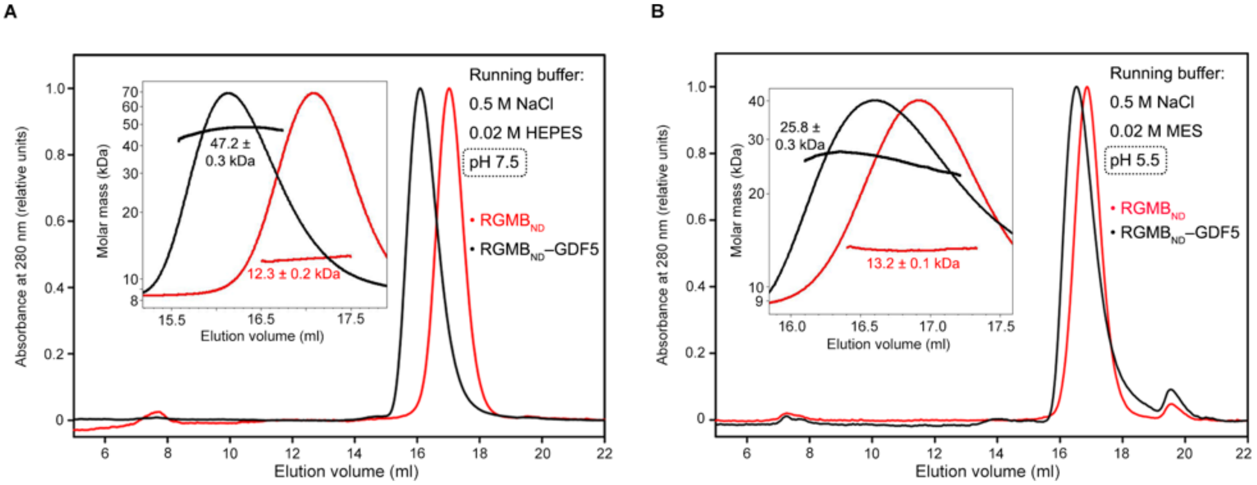

Fig. S10. Size-exclusion chromatography-multi-angle light scattering (SEC-MALS) analysis of RGMB$_{ND}$ and RGMB$_{ND}$ in complex with GDF5.

A, SEC-MALS of RGMB$_{ND}$ (red trace) and RGMB$_{ND}$ in complex with GDF5 (black trace) at pH 7.5. **B**, SEC-MALS of RGMB$_{ND}$ (red trace) and RGMB$_{ND}$ in complex with GDF5 (black trace) at pH 5.5. Traces of absorbance at 280 nm are shown as continuous lines. Insets show peak regions with molecular weight values (with associated statistical uncertainties, calculated by the Astra software (Wyatt Technologies)) were measured at the peak of the refractive index traces: RGMB$_{ND}$ (12.3 ± 0.2 kDa) and RGMB$_{ND}$–GDF5 (47.2 ± 0.3 kDa) at pH 7.5 (**A**), RGMB$_{ND}$ (13.2 ± 0.1 kDa) and RGMB$_{ND}$–GDF5 (25.8 ± 0.3 kDa) at pH 5.5 (**B**). Theoretical molecular weights: RGMB$_{ND}$, 10.5 kDa; dimeric GDF5, 27.2 kDa; RGMB$_{ND}$:GDF5 2:2 mol:mol, 48.2 kDa; RGMB$_{ND}$:GDF5 1:2 mol:mol, 37.7 kDa. RGMB$_{ND}$ was enzymatically deglycosylated with Endoglycosidase F1 leaving one N-acetyl-D-glucosamine moiety on Asn120 of RGMB (0.2 kDa) (**Fig. 1F**), GDF5 did not contain any glycans.

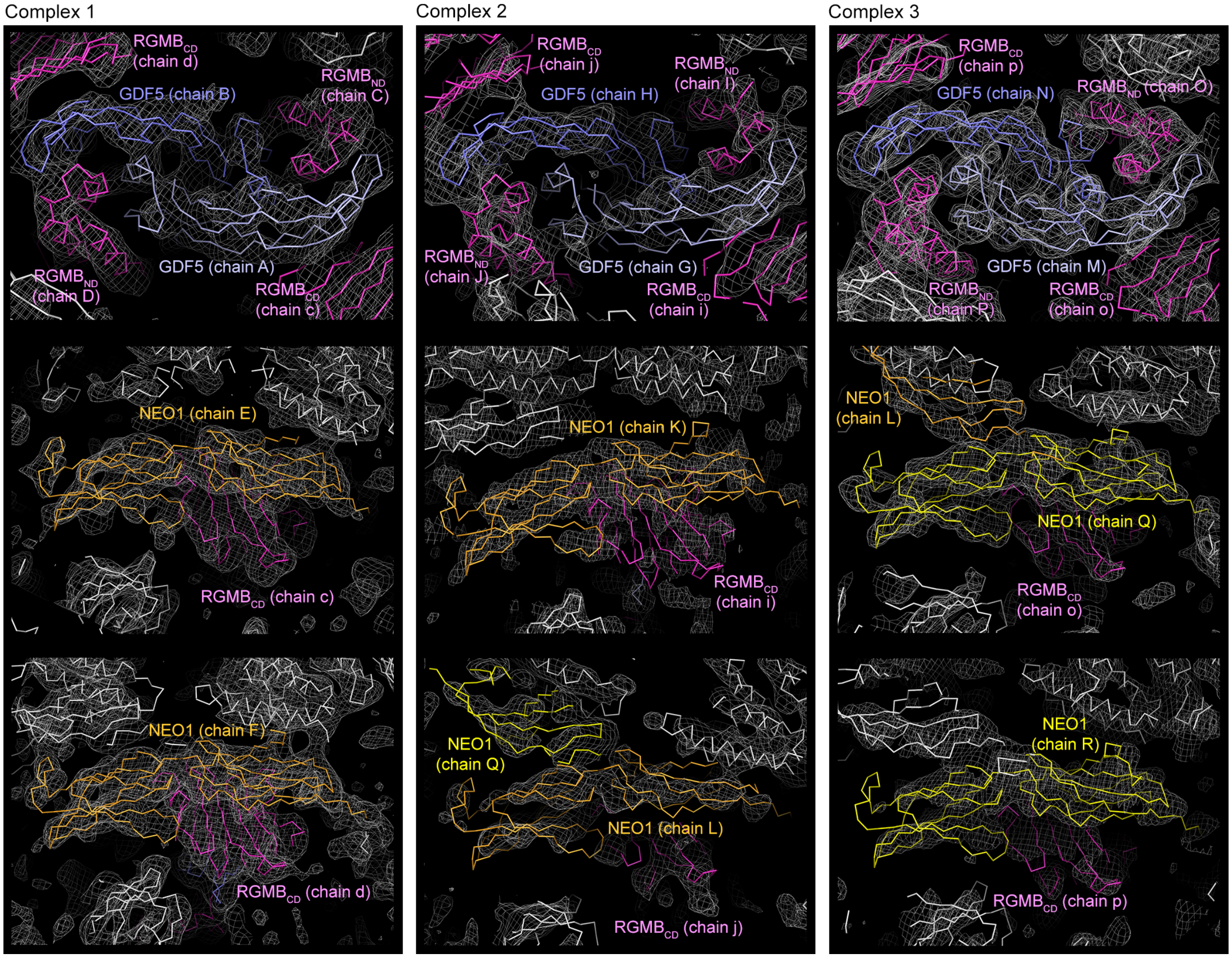

Fig. S11. Electron density map of the NEO1–RGMB–GDF5 ternary complexes at 5.5 Å resolution.

$2F_o$-F_c electron density maps were calculated using phenix.refine and visualized as white mesh at 1.0 σ contour level in PyMOL. The Cα backbones of the three NEO1–RGMB–GDF5 complexes in the asymmetric unit are shown as colored ribbons (GDF5, light blue; RGMB, pink; NEO1, yellow). The Cα backbones of symmetry-related molecules are shown in light gray. Labelling of protein chains corresponds to Materials and Methods.

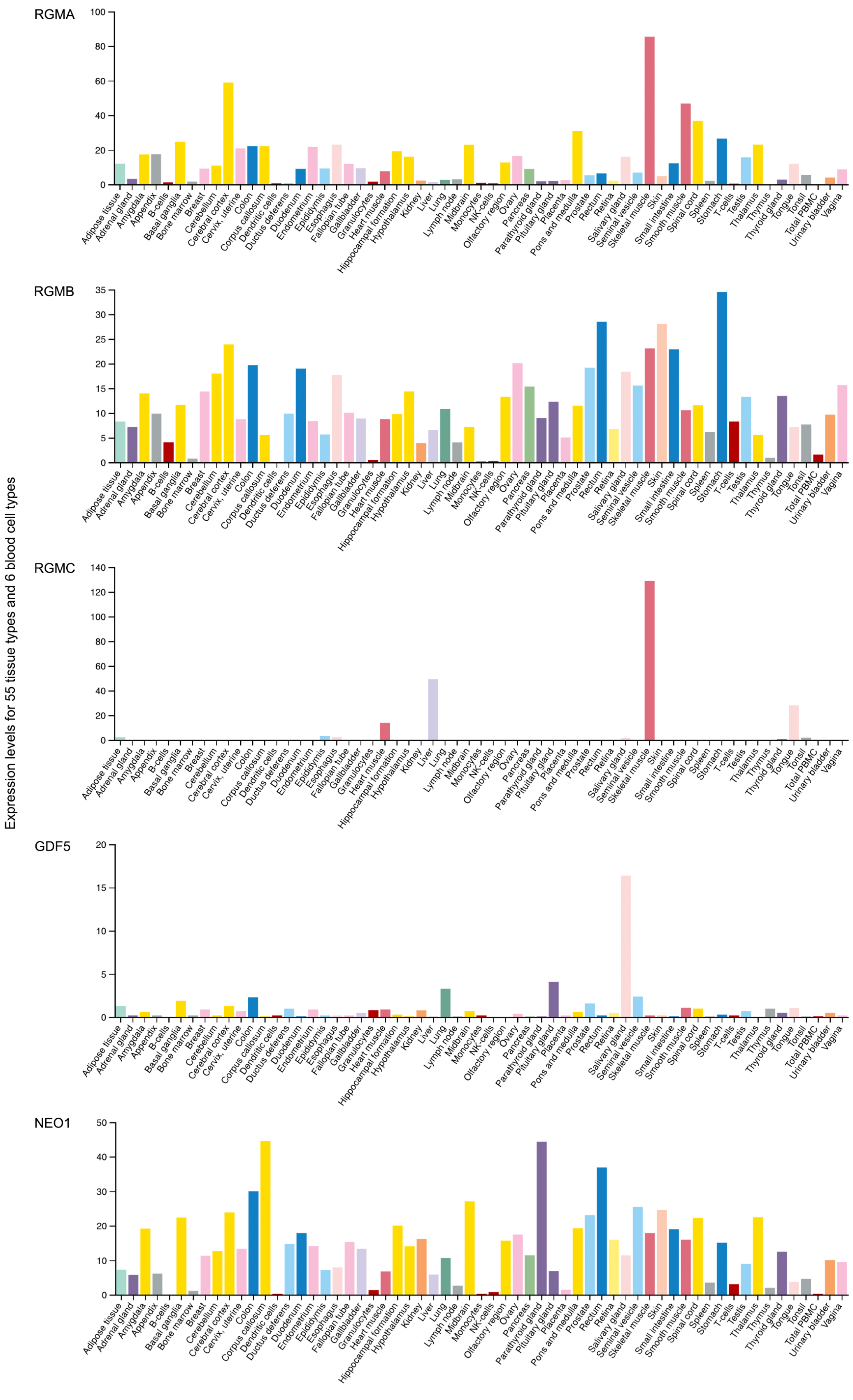
RGMA
RGMB
RGMC
GDF5
NEO1
Expression levels for 55 tissue types and 6 blood cell types
Adipose tissue
Adrenal gland
Amygdala
Appendix
B-cells
Basal ganglia
Bone marrow
Breast
Cerebellum
Cerebral cortex
Cervix, uterine
Colon
Corpus callosum
Dendritic cells
Ductus deferens
Duodenum
Endometrium
Epididymis
Esophagus
Fallopian tube
Gallbladder
Granulocytes
Heart muscle
Hippocampal formation
Hypothalamus
Kidney
Liver
Lung
Lymph node
Midbrain
Monocytes
NK-cells
Olfactory region
Ovary
Pancreas
Parathyroid gland
Pituitary gland
Placenta
Pons and medulla
Prostate
Rectum
Retina
Salivary gland
Seminal vesicle
Skeletal muscle
Skin
Small intestine
Smooth muscle
Spinal cord
Spleen
Stomach
T-cells
Testis
Thalamus
Thymus
Thyroid gland
Tongue
Tonsil
Total PBMC
Urinary bladder
Vagina

Fig. S12. Expression levels of three human RGMs, GDF5 and NEO1 in various tissues.

Consensus normalized expression levels of three RGMs, GDF5 and NEO1 in 55 tissue types and 6 blood cell types, created by combining the data from the three transcriptomics data sets (HPA, GTEx and FANTOM5) (https://www.proteinatlas.org; (24)). Color coding is based on tissue groups, each consisting of tissues with functional features in common.

Supplementary References

1. A. R. Aricescu, W. Lu, E. Y. Jones, A time- and cost-efficient system for high-level protein production in mammalian cells. *Acta Cryst D* **62**, 1243–1250 (2006).

2. B. Demeneix *et al.*, Gene transfer with lipospermines and polyethylenimines. *Adv Drug Deliv Rev* **30**, 85–95 (1998).

3. D. Logeart-Avramoglou, M. Bourguignon, K. Oudina, P. Ten Dijke, H. Petite, An assay for the determination of biologically active bone morphogenetic proteins using cells transfected with an inhibitor of differentiation promoter-luciferase construct. *Anal Biochem* **349**, 78–86 (2006).

4. Y. Cheng, W. H. Prusoff, Relationship between the inhibition constant (K_I) and the concentration of inhibitor which causes 50 per cent inhibition (I_{50}) of an enzymatic reaction. *Biochem Pharmacol* **22**, 3099–3108 (1973).

5. E. G. Healey *et al.*, Repulsive guidance molecule is a structural bridge between neogenin and bone morphogenetic protein. *Nat Struct Mol Biol* **22**, 458–465 (2015).

6. K. R. MacKenzie, J. H. Prestegard, D. M. Engelman, A transmembrane helix dimer: structure and implications. *Science* **276**, 131–133 (1997).

7. S. Harth, A. Kotzsch, J. Hu, W. Sebald, T. D. Mueller, A selection fit mechanism in BMP receptor IA as a possible source for BMP ligand-receptor promiscuity. *PLoS one* **5** (2010).

8. A. Kotzsch, J. Nickel, A. Seher, W. Sebald, T. D. Muller, Crystal structure analysis reveals a spring-loaded latch as molecular mechanism for GDF-5-type I receptor specificity. *EMBO J* **28**, 937–947 (2009).

9. UniProt Consortium, UniProt: the universal protein knowledgebase. *Nucleic Acids Res* **46**, 2699 (2018).

10. J. Pei, B. H. Kim, N. V. Grishin, PROMALS3D: a tool for multiple protein sequence and structure alignments. *Nucleic Acids Res* **36**, 2295–2300 (2008).

11. S. Keller, J. Nickel, J. L. Zhang, W. Sebald, T. D. Mueller, Molecular recognition of BMP-2 and BMP receptor IA. *Nat Struct Mol Biol* **11**, 481–488 (2004).

12. G. P. Allendorph, M. J. Isaacs, Y. Kawakami, J. C. Izpisua Belmonte, S. Choe, BMP-3 and BMP-6 structures illuminate the nature of binding specificity with receptors. *Biochemistry* **46**, 12238–12247 (2007).

13. S. Saremba *et al.*, Type I receptor binding of bone morphogenetic protein 6 is dependent on N-glycosylation of the ligand. *FEBS J* **275**, 172–183 (2008).

14. D. L. Griffith, P. C. Keck, T. K. Sampath, D. C. Rueger, W. D. Carlson, Three-dimensional structure of recombinant human osteogenic protein 1: structural paradigm for the transforming growth factor beta superfamily. *Proc Natl Acad Sci USA* **93**, 878–883 (1996).

15. Z. Wei, R. M. Salmon, P. D. Upton, N. W. Morrell, W. Li, Regulation of bone morphogenetic protein 9 (BMP9) by redox-dependent proteolysis. *J Biol Chem* **289**, 31150–31159 (2014).

16. A. K. Padyana *et al.*, Crystal structure of human GDF11. *Acta Crystallogr F* **72**, 160–164 (2016).

17. J. Y. Hsu *et al.*, Non-homeostatic body weight regulation through a brainstem-restricted receptor

for GDF15. *Nature* **550**, 255–259 (2017).

18. C. S. Bond, A. W. Schuttelkopf, ALINE: a WYSIWYG protein-sequence alignment editor for publication-quality alignments. *Acta Cryst D* **65**, 510–512 (2009).

19. T. A. de Beer, K. Berka, J. M. Thornton, R. A. Laskowski, PDBsum additions. *Nucleic Acids Res* **42**, D292–296 (2014).

20. E. Krissinel, K. Henrick, Inference of macromolecular assemblies from crystalline state. *J Mol Biol* **372**, 774–797 (2007).

21. J. Nickel, A. Kotzsch, W. Sebald, T. D. Mueller, A single residue of GDF-5 defines binding specificity to BMP receptor IB. *J Mol Biol* **349**, 933–947 (2005).

22. P. Seemann *et al.*, Activating and deactivating mutations in the receptor interaction site of GDF5 cause symphalangism or brachydactyly type A2. *J Clin Invest* **115**, 2373–2381 (2005).

23. U. Klammert *et al.*, GDF-5 can act as a context-dependent BMP-2 antagonist. *BMC Biol* **13**, 77 (2015).

24. M. Uhlen *et al.*, Proteomics. Tissue-based map of the human proteome. *Science* **347**, 1260419 (2015).
